# Sodium alginate piezoelectric hydrogel loaded with extracellular vesicles derived from bone marrow mesenchymal stem cells promotes repair of Achilles tendon rupture

**DOI:** 10.1186/s12951-025-03606-5

**Published:** 2025-10-01

**Authors:** Ao Duan, Bingqing Lin, Zhencheng Xiong, Xiaolong Shao, Wenzheng Liu, Renliang Zhao, Xiangtian Deng, Chaoyi Zhang, Dong Wang, Zilu Ge, Xiaoran Hu, Wei Lin, Shouye Hu, Guanglin Wang

**Affiliations:** 1 Trauma Medical Center, Department of Orthopedics Surgery, West China Hospital, Sichuan University, Chengdu, 610041 China; 2 Department of Orthopedics, Orthopedic Research Institute, West China Hospital, Sichuan University, Chengdu, 610041 China; 3 College of Materials and Energy, University of Electronic Science and Technology of China, Chengdu, Sichuan 611731 China; 4 Department of Joint Surgery, Xi’an Honghui Hospital, Xi’an Jiaotong University, Xi’an, Shaanxi 710054 China; 5 Department of Gynecology, West China Second Hospital, Sichuan University, Chengdu, 610041 China

**Keywords:** Achilles tendon rupture, SPH-EVs, Repair, Motion monitoring, Sodium alginate piezoelectric hydrogel

## Abstract

Accelerated repair of Achilles tendon rupture and prevention of re-rupture continue to pose significant technical challenges in orthopedic surgery and rehabilitation. Extracellular vesicles (EVs) derived from bone marrow mesenchymal stem cells exhibit substantial therapeutic potential for various degenerative diseases and tissue regeneration. However, the use of EVs alone for repairing ruptured Achilles tendons requires multiple invasive administrations, such as repeated injections, to maintain a therapeutic effect, which increases patient discomfort and the risk of infection. In this study, we innovatively combined EVs with sodium alginate-based piezoelectric hydrogel (SPH) to develop SPH-EVs. By leveraging the slow degradation of SPH in vivo, SPH-EVs enable sustained-release of EVs while generating electrical stimulation, ensuring that an effective therapeutic concentration is maintained at the Achilles tendon fracture site. Additionally, the integrated near-field communication (NFC) module within SPH-EVs allows for real-time monitoring of rehabilitation exercise intensity in the affected area, guiding patients to conduct rehabilitation training within a safe range and minimizing the risk of re-rupture.

**Graphical Abstract:**

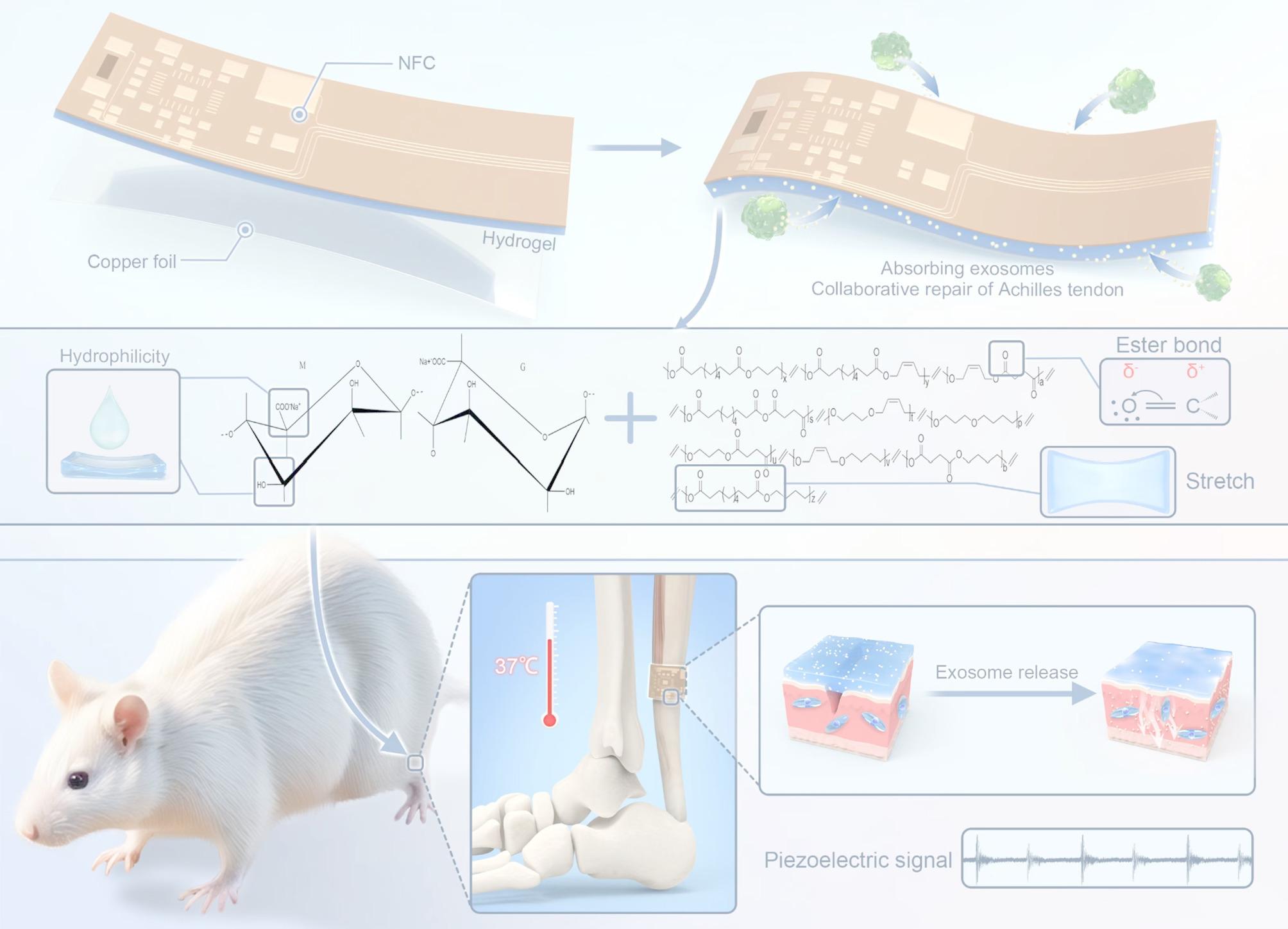

**Supplementary Information:**

The online version contains supplementary material available at 10.1186/s12951-025-03606-5.

## Introduction

The tendon is a soft tissue with high tensile strength, transmitting the mechanical stress of muscle contraction to the skeleton, serving as the fundamental bridge connecting the calcaneus and muscle for complex movements and functions [1]. Unfortunately, Achilles tendon rupture is one of the most common musculoskeletal injuries. In the general population, nearly 5–50 individuals per 100,000 are affected [2, 3]. Surgical treatment involving direct end-to-end repair using sutures and biological or synthetic grafts is considered as the preferred approach to realize tendon rupture repair. However, these repairs often fall short of fully restoring tendon functionality. Meanwhile, due to the invasiveness and limitations of surgical procedures, even complete restoration of tendon function post-surgery exists a risk of re-rupture ranging from 1.7 to 5.6% [4–7]. The prolonged postoperative recovery process may lead patients to prematurely bear weight, and a lack of monitoring and guidance during weight-bearing may further increase the risk of tendon re-rupture [8]. Subsequent surgery for Achilles tendon re-rupture significantly prolongs the recovery time and worsens the prognosis [9]. Therefore, finding a method to aid in the rapid repair of Achilles tendon rupture and reduces the risk of re-rupturing the Achilles tendon is crucial.

Due to the lack of physiological properties of blood vessels in the Achilles tendon tissue, the recovery time of Achilles tendon rapture is often longer than that of normal tissue. In order to speed up the repair of Achilles tendon tissue after surgery, scientists have made many attempts. Recent studies have highlighted the therapeutic potential of mesenchymal stem cells (MSCs) transplantation for various degenerative diseases [10–12]. However, the direct application of stem cells is limited due to potential risk factors, including chromosomal variations and immune rejection [13, 14]. In recent years, growing evidence has indicated that the therapeutic efficacy of MSC-based therapy can be attributed to the paracrine activity of extracellular vesicles (EVs), which transport specific substances to recipient cells [15–17]. Notably, the use of these EVs has shown minimal side effects, such as immunogenicity or tumorigenicity [18–23]. However, due to the rapid clearance and circulation of body fluids, extracellular vesicles are difficult to release continuously and slowly at a specific site, so the extracellular vesicles at the affected area need to be continuously supplemented by invasive means such as injection to maintain the optimal therapeutic concentration [24, 25]. In order to solve this problem, many studies have combined extracellular vesicles with hydrogels to achieve the effect of slow-release extracellular vesicles, and have shown surprising therapeutic effects in the treatment of many diseases [26–29]. However, the existing hydrogel materials show only functions of slow release of EVs, which will undoubtedly greatly weaken the ability of endoplant to repair the Achilles tendon tissue by unit mass.

Many studies have confirmed that the external direct electric stimulation have significant potential in mediating cell migration, promoting collagen synthesis, nerve injury repair, and inducing successful wound [30–39]. However, the portable and operational inconvenience of the devices for external direct electric stimulation has limited their application in tissue injury repair. In recent years, piezoelectric materials were found to provide convenient and effective in-situ electrical stimulation by converting ambient mechanical energy into electric charges, addressing the aforementioned limitations and expanding the application of electrical stimulation in tissue injury repair scenarios. Studies have indicated that tenocytes are sensitive to electrical stimulation, leading to increased proliferation rates and promoting the expression of tenomodulin in tenocytes [40–42]. Thus, a piezoelectric hydrogel seems ideal alternative to realize both slow release or EVs and provide in-situ electric stimulations, both of which is beneficial to improve repair of Achilles tendon. Meanwhile, considering the excellent stress sensing of piezoelectric materials, it can realize synchronous motion monitoring function during the repair of Achilles tendon to monitor the intensity of rehabilitation training in real time and reduce the risk of secondary rupture of Achilles tendon tissue caused by excessive intensity of rehabilitation training. Thus, a piezoelectric materials with good piezoelectric properties, high biocompatibility, complete degradation and drug slow release function for the repair of injured Achilles tendon tissue is highly desired. Therefore, it is urgent to develop piezoelectric materials with good piezoelectric properties, high biocompatibility, complete degradation and drug slow release function for the repair of injured Achilles tendon tissue [43–45].

The hypothesis of this study is that a sodium alginate piezoelectric hydrogel (SPH) was obtained by incorporating sodium alginate into a piezoelectric elastomer matrix to repair tendon rupture. Due to the water absorption characteristics of sodium alginate, SPH has a high water absorption capacity to load large amounts of extracellular vesicles, which become SPH-EVs, and continues to slowly release extracellular vesicles as SPH-EVs slowly degrades in vivo. We speculate that SPH-EVs had positive effect on the repair of Achilles tendon rupture in rats through combination of electric stimulations and the function of extracellular vesicles to promote the repair and regeneration of damaged tissues. In addition, we can combine the NFC module, which monitors the intensity of the Achilles tendon movement in real time, with SPH-EVs, and when implanted in the body, it can reduce the risk of the Achilles tendon breaking again due to excessive exercise intensity. Thus, SPH-EVs will undoubtedly provide us with a promising direction for the treatment of human Achilles tendon rupture repair.

## Materials and methods

### Synthesis of SPH and SPH-EVs

The synthesis of SPH includes two steps, the first step is the synthesis of Bing Qing Piezoelectric Rubber (BQPR) elastomer. Based on the previous research results, we made appropriate adjustments to the components of the piezoelectric elastomer in the previous study to meet the material requirements of this research, and obtained a piezoelectric material - BQPR elastomer - with good piezoelectric performance, high biocompatibility and good mechanical properties. The principle of its piezoelectric generation is consistent with previous studies [39, 46–48]. The BQPR elastomer is synthetized using 1,3-propanediol, 1,4-butanediol, succinic acid, sebacic acid and maleic acid as monomers. The molar ration of 1,4-butanediol/1,3-propanediol is fixed at 1:1. The molar ration of succinic acid/ sebacic acid is fixed at 7:3. The molar ratio of hydroxyl/carboxyl group is set as 1.1:1, and the molar ratio of maleic acid /carboxyl group is set as 1:10, respectively. The mixture was added and heated under nitrogen atmosphere. The reaction is performed at 180°C for 2 h. Then, the catalyst Tetrabutyl titanate (TBT) and the inhibitor tetramethylpiperidine nitrogen oxyfree phosphorous (0.1 wt% each) was added and the mixture is heated to 220 ℃ under reduced pressure (≤ 300 Pa) for 8 h. Finally, the reactants were precipitated and dissolved in chloroform by adding excess methanol to remove the unreacted monomers and oligomers, and the precipitation samples were collected and dried at 60°C to obtain BQPR elastomers. Then, the sodium alginate powder, BQPR elastomer and 0.5wt % dicumyl peroxide (DCP) were mixed in the internal mixer at 120°C according to certain mass proportion for 30 min. The blends were hot-pressed at 10 MPa and 130°C for 10 min. The FPC board with NFC module was gold-plated with 9–10 mA current for 100 s using ion sputtering instrument, and then the sensor was fixed on the BQPR piezoelectric elastomer to prepare SPH composite material. The SPH-EVs was obtained by soaking the SPH in EVs solution for 4 h.

### Characterization and measurement of SPH

Fourier infrared spectroscopy (Thermo Scientific Nicolet iS5, USA) was used to analyze the composition and structure of the samples. X-ray polycrystalline diffraction (Rigaku Ultima IV, Japan) was used to analyze the crystal structure of the samples. The thermal properties of the samples were analyzed using a differential scanning calorimeter (Mettler DSC3, Switzerland). The morphology of the samples was analyzed by scanning electron microscopy (ZEISS, Germany). The mechanical properties were measured by MTS universal testing machine (German AHX850). The contact angle is measured by a surface tension measuring instrument (Lauda Scientific LSA100, Germany).

In the swelling experiment, the sample with a length of 30 mm and a thickness of 1 mm was completely immersed in distilled water, the initial mass G0 of the sample was recorded. The excess water on the surface was wiped out and dried at certain time, and the mass is weighed as G1. The swelling rate was calculated according to formula 1.

1 $$\:\text{S}\text{w}\text{e}\text{l}\text{l}\text{i}\text{n}\text{g}\:\text{r}\text{a}\text{t}\text{e}=\frac{\text{G}1-\text{G}0}{\text{G}0}\text{x}100\text{\%}$$

In the degradation experiment, the circular sample with a radius of 5 mm and a thickness of 1 mm was prepared with the initial mass G0. The sample is soaked in 20 ml PBS phosphate buffer, the excess PBS solution on the surface was wiped out and dried at certain time, and the mass of the sample was weighed as G1. The degradation rate was calculated according to formula 2.

2 $$\:\text{W}\text{e}\text{i}\text{g}\text{h}\text{t}\:\text{l}\text{o}\text{s}\text{s}=\frac{\text{G}0-\text{G}1}{\text{G}0}\text{x}100\text{\%}$$

### Fabrication of NFC devices

After heating the SPH with a size of 1 × 1 cm^2^ at 60℃ for 15 min, it was attached to the plate of the wireless module (1 × 2 cm^2^) (Shenzhen Jialichuang Technology Group Co., Ltd.China) to make the SPH sensor. The sensor was wound around the Achilles tendon as designed and sewed and encapsulated with PDMS film. The piezoelectric signals are collected by SPH sensors and displayed on the remote APP.

### Piezoelectricity test

The sensor’s piezoelectric performance was evaluated using NI DAQExpress software, a computer, and a piezoelectric acquisition card; the piezoelectric response signal was generated by dropping a 15 g insulated ceramic sphere from a height of 15 cm onto a sample secured to the test rig.

### Isolation and culture of tenocytes

Isolation of rat tenocytes as previously described [51]. In this study, we rinsed the obtained rat Achilles tendon tissue 3 times with PBS solution containing antibiotics, and then cut the tissue into small pieces with medical scissors. The Achilles tendon tissue was transferred to a complete medium containing 2 mg/ml collagenase type I (Sigma-Aldrich) and incubated for 2.5 h to allow digestion and separation of the tenocytes. Isolated tenocytes were cultured in Dulbecco’s Modified Eagle Medium (DMEM) (Gibco) with 10% fetal bovine serum (FBS) and 1% penicillin-streptomycin (Gibco). Then change the medium every 3 days. When the cells have reached about 80% fusion, cells were digested by trypsin-EDTA solution (Sigma-Aldrich) and seeded onto plates as passage 0 (P0) cells. P3 cells were used for all subsequent experiments.

### Proliferation test of tenocytes

First, according to the experimental design, 3 groups of tenocytes were cultured using ordinary medium, medium soaked in PDMS and medium soaked in SPH. After 24 h, the experiment was continued according to BeyoClick™EdU-488 Cell Proliferation Assay Kit (Beyotime). First, the tenocytes are digested with trypsin to obtain suspension cells. According to BeyoClick™EdU-488 cell proliferation assay kit, the proliferation rate of tenocytes in each group was measured by flow cytometry. Not only that, we also used the BeyoClick™EdU-594 Cell Proliferation Detection Kit (Beyotime) to treat the cells attached to the wall, and used the fluorescence microscope to directly observe the cell proliferation, so that the proliferation results were more intuitive.

### Apoptosis assay of tenocytes

As described above, tenocytes were cultured using ordinary medium, medium soaked in Polydimethylsiloxane (PDMS) and medium soaked in SPH. After 24 h, the tenocytes were digested with trypsin to obtain suspension cells. The apoptosis rate of chondrocytes in each group was detected by flow cytometry according to the instructions of the Annexin V-FITC/PI Apoptosis Detection Kit (Vazyme). To ensure the objectivity and reliability of the results, tenocytes in each group were evaluated using the TUNEL Bright Green Apoptosis Detection Kit (Vazyme). According to the kit’s instructions, TUNEL-positive cells in each group were identified, observed, and photographed under a fluorescence microscope.

### Cell cytotoxicity test

As described above, 3 groups of tenocytes were cultured using ordinary medium, medium soaked in PDMS and medium soaked in SPH. The cell cytotoxicity in each group was assessed using a live/dead assay kit (Abbkine). Cells were cultured for 3 days, then treated with a solution containing Calcein AM and PI at 37°C for 30 min, following the manufacturer’s instructions. Live cells emitting green fluorescence and dead cells emitting red fluorescence were visualized using laser scanning confocal microscopy (Nikon).

### Cell morphology staining

As described above, 3 groups of tenocytes were cultured using ordinary medium, medium soaked in PDMS and medium soaked in SPH. Cells were cultured for 24 h, the Cell morphology of tenocytes in each group was detected by fluorescence microscope according to the instructions of the Phalloidin Dyeing kit (Invitrogen).

### Migration assay of tenocytes

As described above, 3 groups of tenocytes were cultured using ordinary medium, medium soaked in PDMS and medium soaked in SPH. After a 24-hour incubation period, tenocytes were fixed using 4% paraformaldehyde for 15 min, then stained with 0.5% crystal violet for 30 min and washed three times with PBS. The top surface of the upper chamber was swabbed to eliminate cells that had not migrated to the lower chamber’s surface. The migration rate of tenocytes in each group was observed under a microscope, with 4 randomly chosen fields analyzed.

### Low intensity pulsed ultrasound (LIPUS) stimulates tenocytes in vitro

Low intensity pulsed ultrasound (LIPUS) was used to induce the generation of electrical signal from SPH/SPH-EVs for in vitro study. Parameters for LIPUS were set as 1 w cm − 2, 20% duty ratio, 1 MHz, 20 min per day.

### Experimental animals and procedures

Male Sprague-Dawley rats weighing 200–220 g were sourced from the Animal Medicine Center at West China Medical College, Sichuan University for this research. The rats were randomly divided into groups, each consisting of at least 10 rats. Anesthesia was induced in all rats via isoflurane inhalation. Achilles tendon transection surgery was performed on the right leg of the rats. In the sham group, a surgical incision was made in the skin and muscle without severing the Achilles tendon. In the control group, the Achilles tendon of all rats was left unsutured after amputation. In other groups, the Achilles tendon was sutured and wrapped with PMDS, SPH, or SPH-EVs, respectively. To manage postoperative pain, all rats received 0.05 mg/kg buprenorphine. Gentamicin at a dose of 5 mg/kg was administered to prevent postoperative infections. All animal procedures were carried out in compliance with the guidelines established by the Animal Care and Use Committee of West China Hospital. The study received approval from the Animal Protection and Ethics Committee of Sichuan University (No.20240517005), and strictly followed the National Research Council’s Guide for the Care and Use of Laboratory Animals. After an 8-week feeding period, behavioral experiments were conducted on the rats in each group, followed by the collection of Achilles tendon tissues for further analysis.

### Open field test

The open field test was employed to assess the functional recovery of rats’ Achilles tendons following various treatments. In this test, rats were placed in a new and quiet environment consisting of a square black plastic open field box measuring 80 cm × 80 cm. The rats were allowed to freely explore the box for 5 min without any disturbance. A digital video camera was used to record the rats’ movements and behavior in the box. All relevant data were collected and analyzed using a behavior computing system (TM-Vision, China). The box was divided into nine areas, and parameters such as the duration of the rats’ movement distance and movement time were analyzed.

### Footprint analysis

After 8 weeks of modeling, footprint analysis was conducted on each rat group following the instrument’s operational instructions (GAT-100, China). By observing changes in rat gait, we can indirectly assess the healing of the Achilles tendon and the restoration of locomotion ability. Our analysis will primarily concentrate on two specific indicators, labeled as the mean velocity of motion and the mean right foot support time.

### Biomechanical test

For each group of rats’ Achilles tendons, the width, thickness, and length of the tendons were first measured using a digital caliper, and the measurements were recorded. On a universal material testing machine, the Achilles tendon’s two ends were clamped using custom fixtures, with a layer of gauze wrapped around the ends to prevent damage from the clamps. The suture site was positioned at the midpoint. The machine was zeroed when the Achilles tendon was completely relaxed, then a preload of 1 N was applied to straighten the tendon, followed by zeroing again. The tensile speed was set at 5 mm/min for the tensile test until the Achilles tendon completely ruptured, with the computer recording the maximum load and elongation at break.

### Histological analysis

Tendon samples obtained after 8 weeks of treatment were collected for histological staining. Following excision, the tendon samples were perfused with 4% paraformaldehyde until they hardened. The tendon samples were dissected to remove the surrounding soft tissue. After decalcification, dehydration, and wax embedding, the tendon samples were sectioned into 5 μm thick sections for subsequent experiments. These sections were subsequently stained with HE, Masson’s trichrome, and immunofluorescence. Three distinct frontal-longitudinal sections of each tissue were examined. Two independent blinded investigators graded the HE staining using a modified Stoll scale as previously outlined. Masson’s trichrome staining was utilized to assess collagen arrangement and expression, while immunofluorescence staining was employed to identify specific protein expression. Primary antibodies used included collagen I antibody (1:500, Abcam, ab270993), collagen III antibody (1:500, Abcam, ab7778), and Tnmd antibody (1:500, Abcam, ab203676). Additionally, the heart, liver, spleen, lung and kidney of rats from every groups at 8 weeks post-treatment were collected for the assessment of biological toxicity.

### Statistical analyses

All experiments were performed in at least three independent biological replicates. Data are shown as mean ± standard deviation. GraphPad software 7.0 and SPSS 19.0 were used for statistical analysis. We used the Student’s t test for two-group comparisons and one-way or two-way ANOVA for more than two-group comparison to calculate the P values. A value of *P* < 0.05 was considered statistically significant.

## Results and discussion

### Results

#### Chemical compositions and crystalline behavior of SPH

**Fig. 1:**
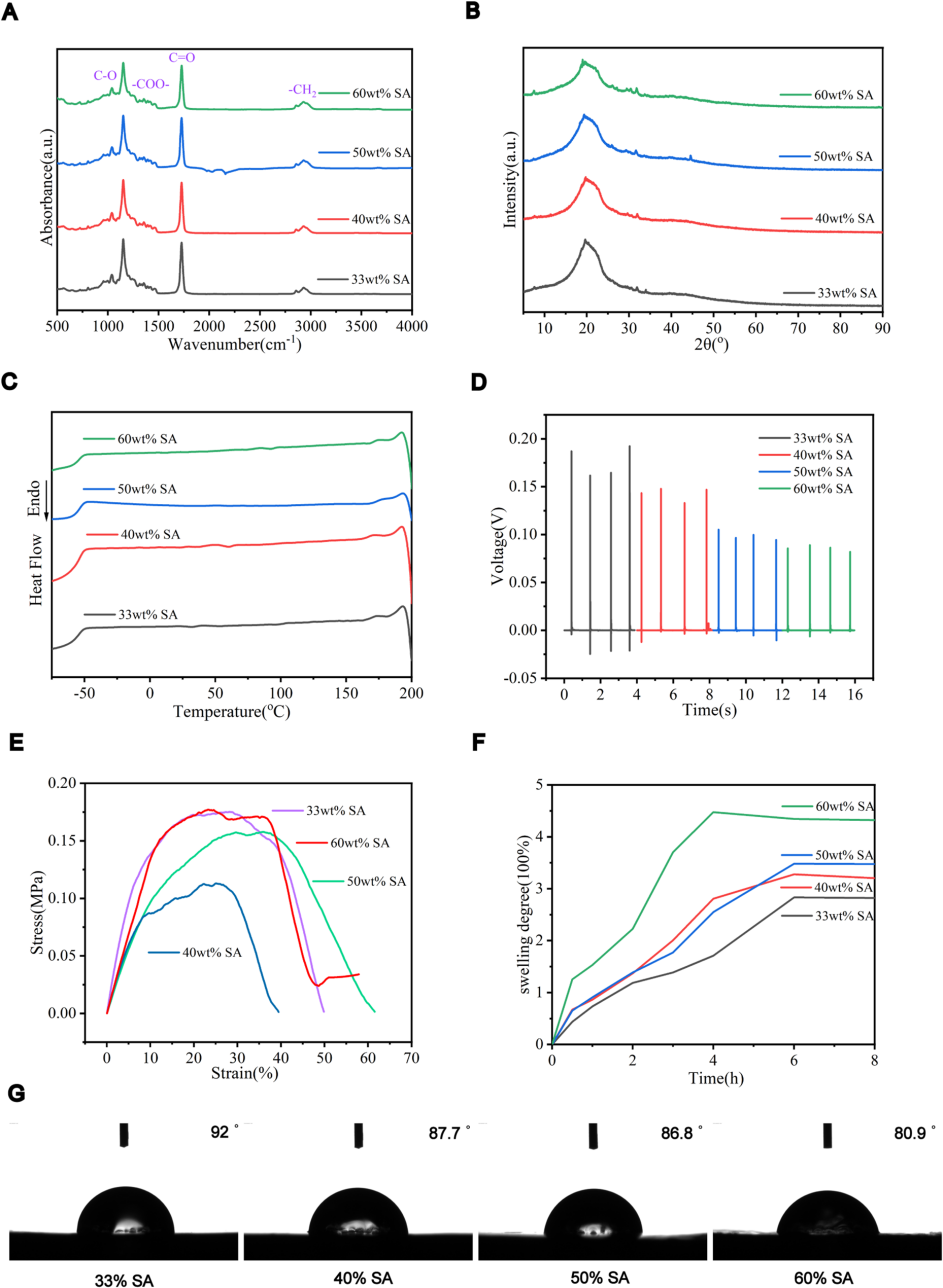
Composition analysis and properties of SPH series. **A**. FTIR spectrum of SPH. **B**. XRD curve of SPH **C**. DSC thermogram of SPH. **D**. Piezoelectric properties of SPH **E**. Stress-strain curve of SPH. **F**. Water absorption of SPH. **G**. Contact angle of SPH

The chemical composition and structure of SPH were characterized by FTIR (Fig. 1A). The absorption peak at 803 cm^− 1^ belongs to the stretching and bending vibration of C = C groups in 1,4-butanediol, the absorption peak at 1038 cm^− 1^ proves the presence of C-O group, and the symmetric stretching vibration of COO- in sodium alginate was found at 1413 cm^− 1^. Meanwhile, the strong vibration peak at 1725 cm^− 1^ belongs to the carbonyl C = O groups in the ester bonds. The characteristic asymmetric and symmetric vibrational absorption peaks at 2853 cm^− 1^ and 2928 cm^− 1^ belongs to the methylene (-CH_2_) groups, respectively. As the SA content in the SPH increases, the peak intensity of the C = O groups decreases and the peak intensity of the C-O, COO- groups increases. These results confirm that sodium alginate is successfully blended in the SPH.

The crystalline behavior of SPH was characterized by XRD (Fig. 1B). In the range of 5°-90°, the diffraction peak intensity at 20° gradually decreased as the increase of SA content, indicate the amorphous nature of the SPH. The thermal change of SPH is represented by the DSC curve (Fig. 1C). With the increase of SA content, the glass transition temperature of BQPR elastomer slightly shifted from − 54.9°C to -54.35°C, and that of sodium alginate slightly shifted from 174.4°C to 173°C, indicating increased compatibility between the SA and the BQPR elastomer phases.

#### Piezoelectric and mechanical performances of SPH

The piezoelectric performance is investigated using a 15 g ceramic insulating ball which was allowed to free fall from a height of 15 cm to the surface of the SPH, and the output piezoelectric voltageof the SPH were collected (Fig. 1D). As the piezoelectricity of SPH is mainly originated from its carbon-oxygen double bonds, therefore, with the increase of SA content, the piezoelectric performance of SPH showed a slightly decrease. However, the SPH-50 still maintained a high piezoelectric output, which provided a good support for the subsequent electrical stimulation treatment of the Achilles tendon.

Mechanical properties are also an important factor limiting Achilles tendon repair. Compatible mechanical properties allow matched stretchable motion of Achilles tendon and SPH. SPH stress-strain curves for different components have been measured (Fig. 1E). The SPH initially exhibits a linear elastic deformation, followed by an obvious plastic deformation until fracture. The SPH-50 shows an elongation at break of 33.88%, which is far high than that of human Achilles tendon (8.8% [49]). Besides, the elastic modulus of SPH-50 is 60.40 MPa. It is much higher than that of reported hydrogels (2–20 MPa), which helps SPH to simulate the dynamic mechanical properties of the Achilles tendon [50].

Water absorption of SPH has also been demonstrated (Fig, 1F), where the hydroxyl and carboxyl groups of the hydrophilic groups of SPH increase as the SA content increases, which further increase its water absorption property. The water absorption of SPH-50 is 300% higher than its initial mass, giving it superior water absorption properties. To further investigate the hydrophilicity of SPH, a contact angle test (Fig. 1G) is performed. The results showed that the contact angle of the SPH decreased with increasing SA content, indicating increased hydrophilicity of SPH. The SPH-50 showed high hydrophilicity. Thus, the SPH-50 seems have optimum performances, which was selected for further analysis.

#### Morphology and properties of SPH

**Fig. 2:**
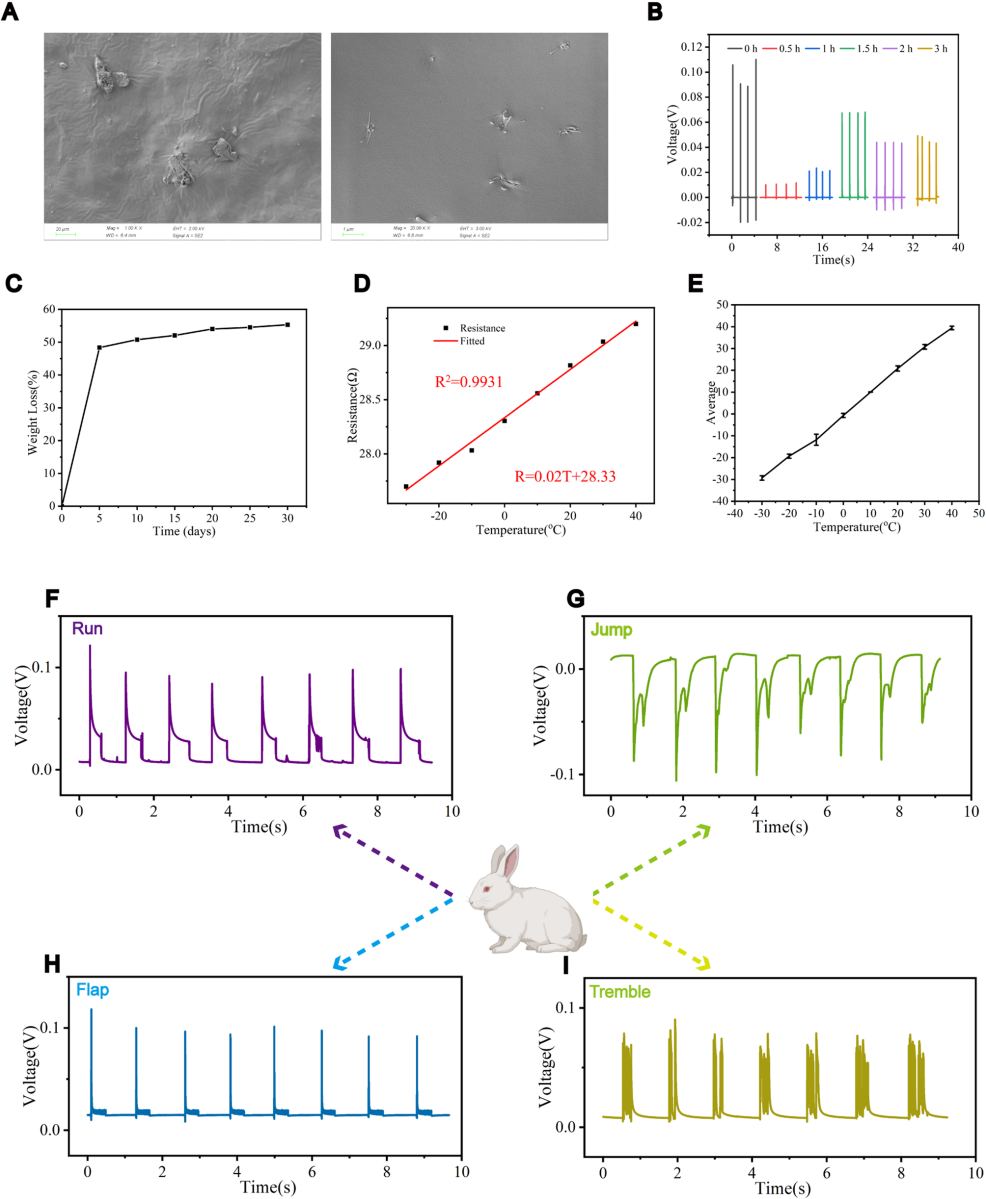
Piezoelectric properties of SPH. **A.** SEM image of SPH surface. **B.** Piezoelectricity curve of SPH after water absorption. **C.** Degradability curve of SPH. **D.** Temperature sensing curve of SPH. **E.** Curve of mean deviation between measured temperature and actual temperature of SPH. **F-I.** The piezoelectric output voltage of sensor under various natural motion states of rabbits in vivo

The morphology of SPH was characterized by SEM. The surface of SPH was smooth, and SA was uniformly distributed on the surface of the composite as columnar fibers (Fig. 2A). The piezoelectric properties of SPH after water absorption were measured (Fig. 2B). The results showed that the piezoelectric properties of SPH initially decreased and then increased, and the piezoelectric output voltage was maintained at about 50 mv, which show a high piezoelectric output to achieve electrical stimulation to repair the Achilles tendon. The degradation performance of SPH was characterized (Fig. 2C). SPH has good degradation performance in vitro, and the rapid degradation of SPH in the first 5 days is mainly due to the rapid degradation of the low molecular weight segments, while the slow degradation subsequently is due to the hydrolysis of ester bonds.

It also monitors a rabbit’s body temperature in real time. The principle is the resistance of metal changes when the temperature varies. The temperature sensing data of the FPC board, the temperature and resistance correspond to each other, and its fitting curve indicates that the temperature and resistance are linear and the fit is greater than 0.99 (Fig. 2D). When comparing the actual temperature with the measured temperature (Fig. 2E), it is confirmed that the measured temperature and the actual temperature error is small, and the temperature sensing is relatively accurate. In order to further investigate the piezoelectric signals of SPH in animals, this study implanted SPH into rabbits and the piezoelectric output when the rabbits in running (Fig. 2F), jumping (Fig. 2G), fluttering (Fig. 2H), and trembling (Fig. 2I) states was recorded. The output voltage exhibits different periodic changes under different movement patterns. Thus, the motion patterns is identified by waveform of piezoelectric voltage.

### Biocompatibility of SPH in vivo and in vitro

An excellent biocompatibility is of great concern for materials implanted in vivo. In order to explore the biocompatibility of SPH in vivo and in vitro, we conducted a series of experiments. First, we cultured rat tenocytes in complete media soaked in SPH and PDMS respectively. As described in the methods section, the proliferation rate and apoptosis rate of tenocytes in each group were measured by cytometry. The results showed that compared with sham group, there was no significant difference in cell proliferation rate and apoptosis rate in SPH group (Supplementary Fig. 1A, B, C and D). In order to explore the toxicity of SPH to tenocytes, the 3 groups of tenocytes cultured for 72 h were stained with a live and dead cell staining kit, and the results showed that the survival rate of the three groups of cells was above 90%, with no significant difference (Supplementary Figs. 1E and F). We also used transwell chamber to verify the effect of SPH on cell migration ability, and the results showed that the cell migration ability of the 3 groups was good without significant difference (Supplementary Figs. 1G and H). In addition, the morphology of tenocytes were staining with phalloidine. The results showed that there was no significant difference in cell morphology among the 3 groups (Supplementary Fig. 1I). Furthermore, HE staining was performed on the heart, liver, spleen, lungs, and kidneys of the rats implanted with SPH, and there was no significant difference in the histological structure of these organs compared with the sham group (Supplementary Figs. 2A, B,C, D and E). These results all confirmed that SPH has excellent biocompatibility in vivo and in vitro.

#### The SPH can significantly improve the functional behavior and motor ability of rats after Achilles tendon injury

**Fig. 3:**
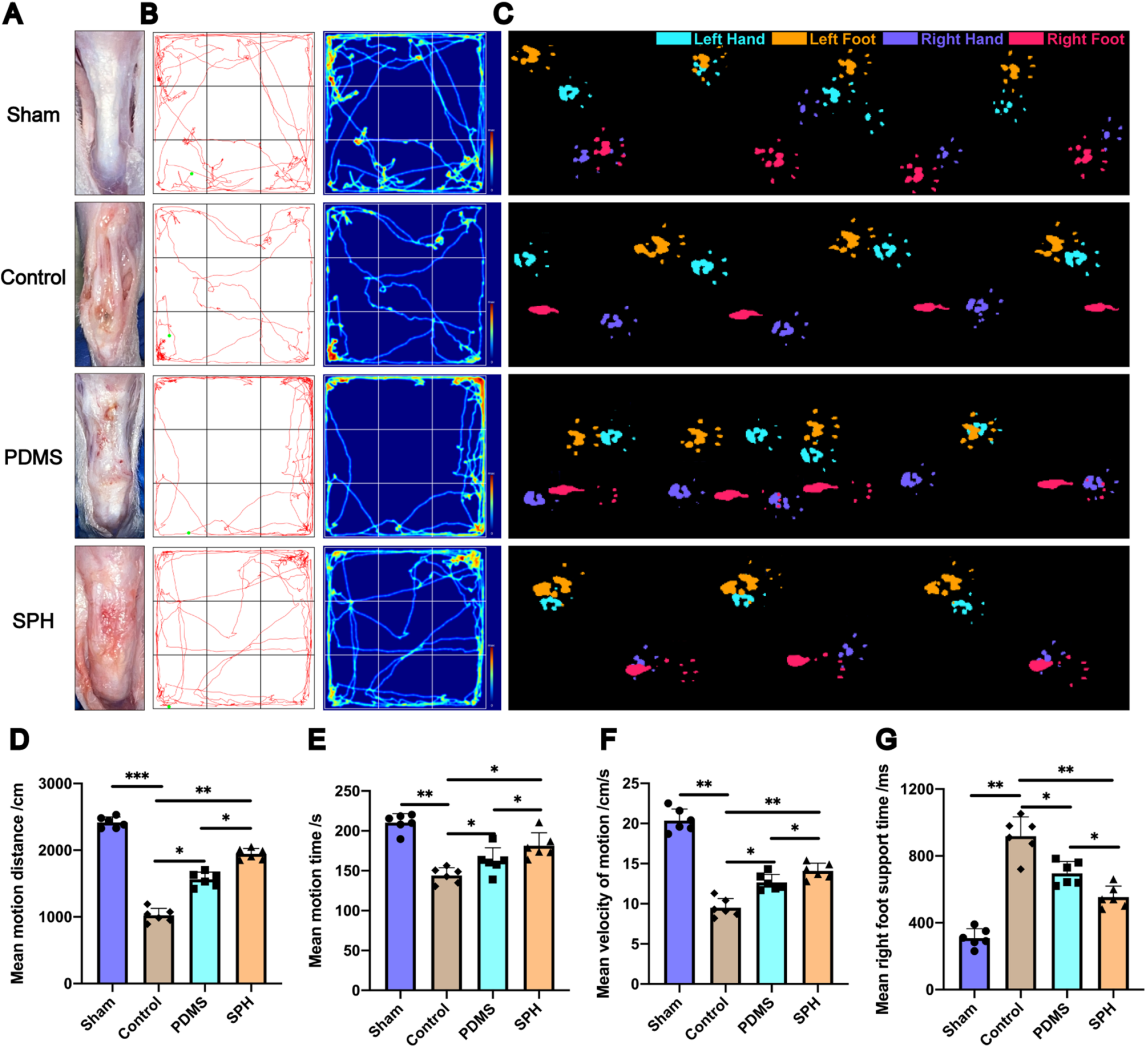
The SPH can significantly improve the functional behavior and motor ability of rats after achilles tendon injury. **A.** General view of the tendon in each group after 8 weeks. **B.** Motion trace and heat map of each group rat in open field test. **C.** The results of footprint analysis in each group of rats. **D**,** E.**The mean motion time and distance of rats in open field test (*n* = 6, one-way ANOVA). **F.** The mean motion speede and right foot support time of rats in footprint analysis (*n* = 6, one-way ANOVA). Data are presented as mean ± SD. *P < 0.05, **P < 0.01, ***P < 0.001

To further evaluate the piezoelectric effect of SPH in vivo, we created a rat model of Achilles tendon repture by completely dissecting and suturing the Achilles tendon using the modified Kessler technique. In order to avoid the interference of material bridging on the tendon, PDMS or SPH is wrapped around the injured tendon to promote tendon healing. The rats in each group were fed for 8 weeks after modeling, and the open field test and the footprint analysis were carried out to evaluate the recovery of Achilles tendon in each group from the perspective of behavior. The Achilles tendon was taken for gross morphological evaluation. The results showed that the Achilles tendon was missing in the Control group, and the broken end healed by scar healing (Fig. 3A). The Achilles tendon healed in both the PDMS group and the SPH group, but there were still more scar tissue in the PDMS group, and the Achilles tendon became shorter and thicker, suggesting that the Achilles tendon in the SPH group had a better recovery (Fig. 3A). The results of open field test showed that compared with the Control group, the average moving distance and the average moving time of the PDMS group were better than those of the Control group (Figs. 3B, D and E). Surprisingly, these indexes of rats in the SPH group were significantly better than those in the PDMS group, and were second only to those in the Sham operation group (Figs. 3B, D and E). Moreover, the results of footprint analysis also showed that the PDMS group rats showed significantly faster gait recovery and improved motor co-ordination compared with the Control group rats, and this favorable effect was increased in the SPH group (Figs. 3C and F). The mean right foot support time of SPH group was also significantly lower than that of PDMS group (Fig. 3G). The above behavioral experimental results showed that SPH significantly improved the exercise ability of rats with Achilles tendon injury from a behavioral perspective.

#### The SPH can significantly promote the repair of Achilles tendon after injury at historical level

**Fig. 4:**
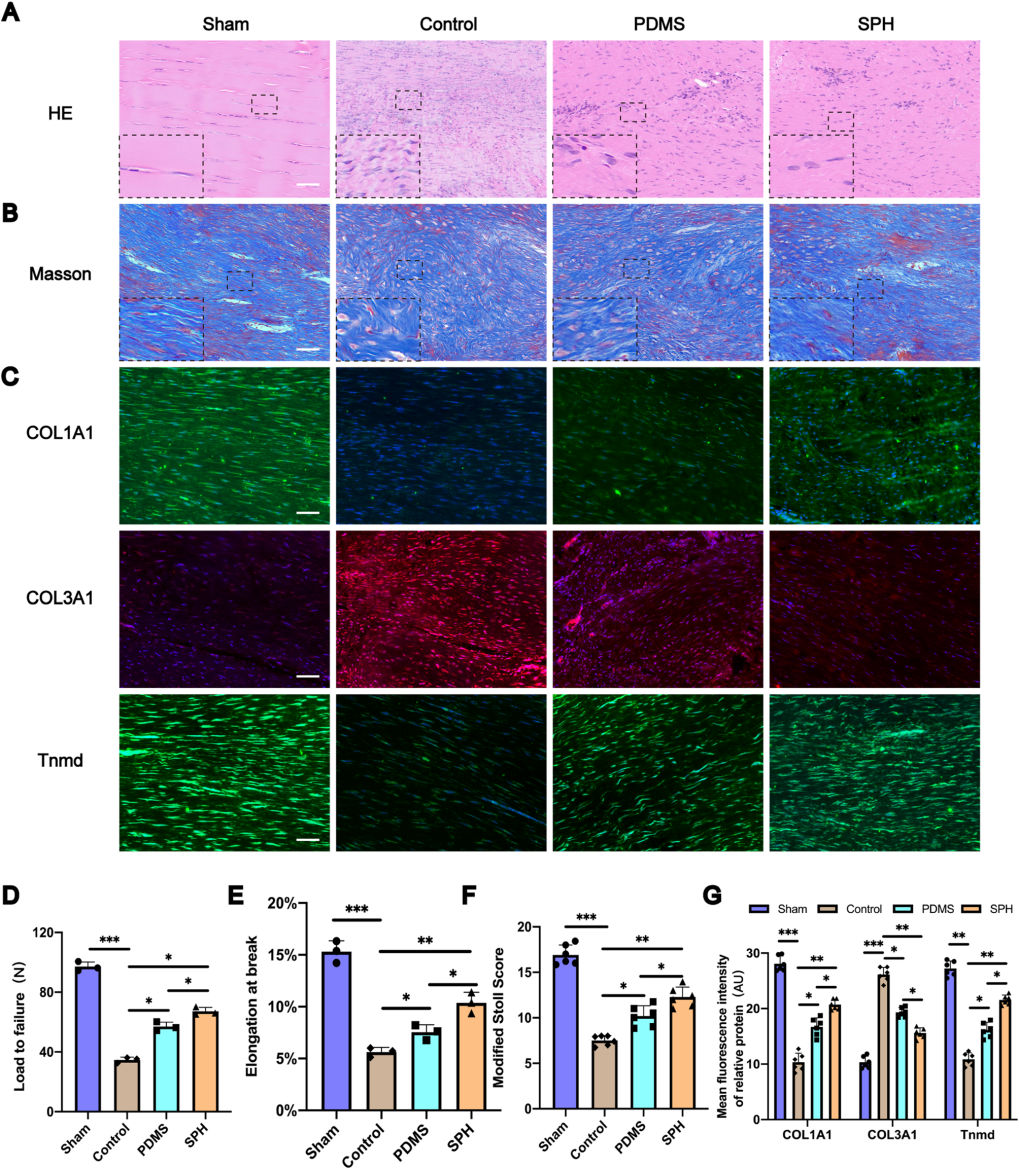
The SPH can significantly promote the repair of achilles tendon after injury at historical level. **A**,** B.** HE staining and Masson staining of the tendon in each group after 8 weeks (Scale Bar = 400 μm). **C.** Expression levels of COL1A1, COL3A1 and Tnmd as determined by immunofluorescence staining (Scale Bar = 400 μm). **D.**Load to failure of Achilles tendon tissue in each group (*n* = 3, one-way ANOVA). **E.** Elongation at break of Achilles tendon tissue in each group (*n* = 3, one-way ANOVA). **F.** Quantitative analysis of HE staining according to the modified Stoll score (*n* = 6, one-way ANOVA). **G.** Quantification of mean fluorescence intensity of COL1A1, COL3A1 and Tnmd in each group (*n* = 6, one-way ANOVA). Data are presented as mean ± SD. *P < 0.05, **P < 0.01, ***P < 0.001

The biomechanical property recovery was essential to evaluate tendon recovery. We conducted biomechanical analysis of the Achilles tendon samples in each group to evaluate the strength of the Achilles tendon after healing. The results showed that compared with the PDMS group, the Achilles tendons in the SPH group had larger load to failure and elongation at break (Figs. 4D and E), indicating that the Achilles tendons in the SPH group had greater strength and better recovery. Next, HE staining and Masson staining were performed on the Achilles tendon tissues of each group. According to HE and Masson staining, the extracellular matrix collagen arrangement of the Achilles tendon in the Control group was disordered, with cell proliferation, scar formation and vascular hyperplasia (Figs. 4A and B). The PDMS group showed similar histological features to the Control group (Figs. 4A and B). However, for the SPH group, the collagen tissue was more orderly with fewer scar and blood vessels (Figs. 4A and B). The modified Stoll score was used to evaluate tendon healing [51, 52]. The modified Stoll score showed higher scores in the SPH group compared to the PDMS group, indicating better healing in the SPH group (Fig. 4F).

In addition, immunofluorescence staining was performed on the Achilles tendons of each group to explore the expression of collagen type I alpha 1 (COL1A1), collagen type III alpha 1 (COL3A1) and tenomodulin (Tnmd) in the Achilles tendons of each group. COL1A1 is the main structural component of tendon tissue, and its high expression indicates better tendon regeneration and healing [53]. Increased expression of COL3A1 means that Achilles tendon repair is hampered [54]. Tnmd is used as a characteristic marker of maturation of tendon-line cells. The higher the expression of Tnmd, the better the cell differentiation [55]. Immunofluorescence staining showed that the expression of COL1A1 and Tnmd was higher and the expression of COL3A1 was lower in SPH group than in PDMS group (Figs. 4C and G). All these results indicated that the healing of Achilles tendon in SPH group was better than that in PDMS group.

#### Identification of extracellular vesicles derived from rat bone marrow mesenchymal stem cells and assembly of the SPH-EVs

**Fig. 5:**
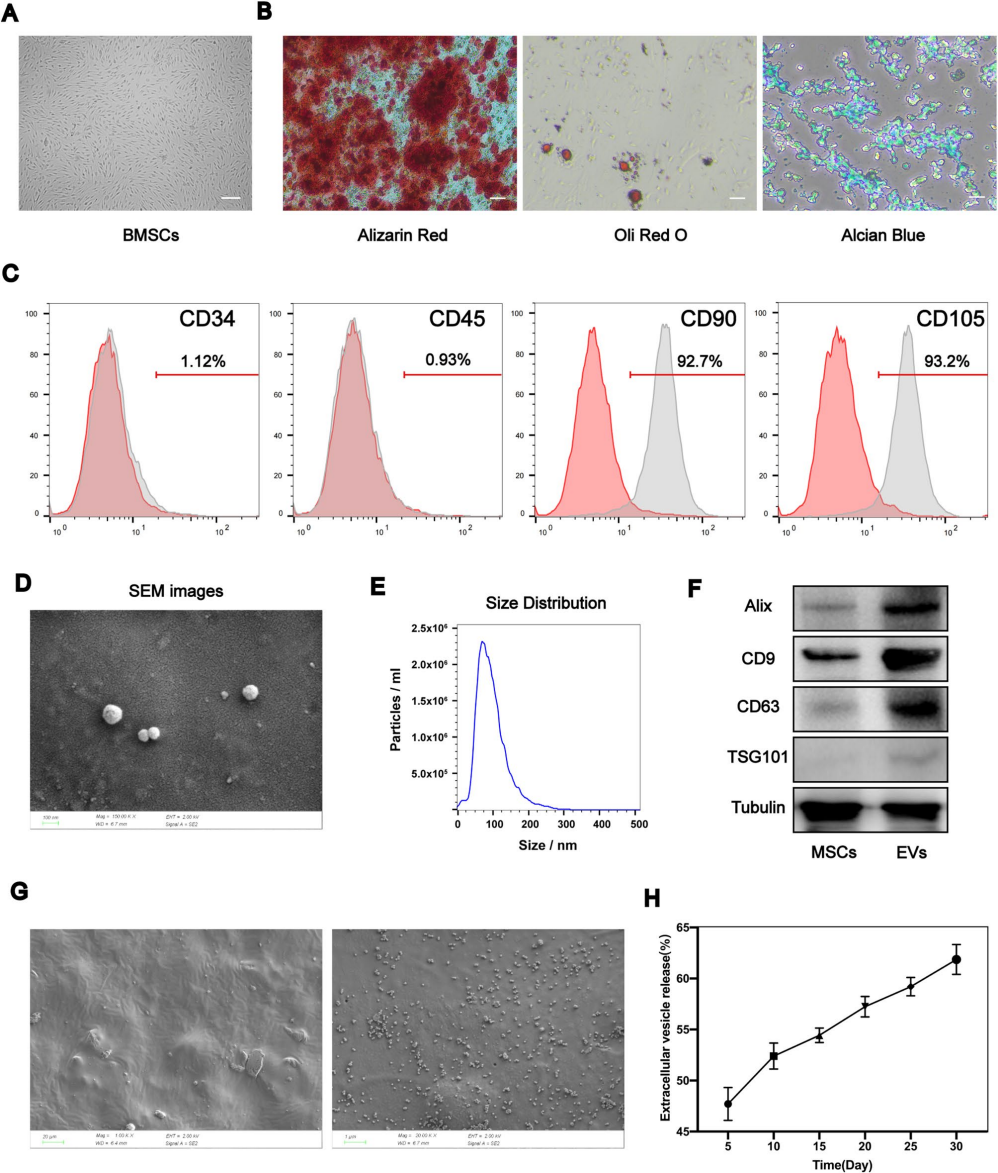
Identification of extracellular vesicles derived from rat bone marrow mesenchymal stem cells and assembly of the SPH-EVs. **A**. BMSCs exhibited a representative spindle-like morphology. **B**. BMSCs show the multidirectional differentiation potential of osteogenesis, adipogenesis, and chondrogenesis. **C**. The characteristic cell surface markers of BMSCs were analyzed by flow cytometry. **D**. SEM images of EVs. **E**. The particle size distributions of EVs were analyzed by NanoSight. **F**. Extracellular vesicles makers (CD9, CD63, Alix, TSG101, Calnexin) analyzed by western blot. **G**. SEM images of SPH-EVs. **H**. The concentration of EVs released by SPH-EVs was determined using a BCA protein assay kit

In accordance with the procedures outlined in the Methods section, bone marrow-derived mesenchymal stem cells (BMSCs) were isolated and subsequently cultured. Upon microscopic observation, the BMSCs exhibited a distinct spindle-shaped morphology (Fig. 5A). Osteogenic, adipogenic, and chondrogenic media were employed to induce and culture the obtained BMSCs. Verification of differentiation towards osteogenesis (Fig. 5B left panel), lipogenesis (Fig. 5B middle panel), and chondrogenesis (Fig. 5B right panels) was conducted using Alizarin Red staining, Oli Red O staining, and Alcian Blue staining, respectively. The investigation revealed the multilineage differentiation potential of the extracted BMSCs, aligning with the characteristic differentiation features of mesenchymal stem cells. Additionally, flow cytometry analysis was performed to assess the surface marker protein expression on the isolated BMSCs, indicating a specific marker profile consistent with MSCs (Fig. 5C). The above results showed that the extracted cells were BMSCs.

We extracted EVs from the supernatant of BMSCs. Initially, the extracted EVs was observed using a scanning electron microscope, revealing a round or disk-shaped morphology (Fig. 5D), consistent with the typical external characteristics of extracellular vesicles. NanoSight was also utilized to analyze the particle size of the extracted EVs, showing that the diameter of EVs mainly ranged from 50 to 200 nm, in line with the characteristic particle size distribution of extracellular vesicles (Fig. 5E). Western blot results indicated the presence of extracellular vesicle markers in EVs but not in BMSCs, including CD9, CD63, Alix, and TSG101 (Fig. 5F). All the aforementioned findings confirmed that the material we isolated was indeed extracellular vesicles.

Due to the good water absorption of SPH, we soaked the EVs solution with SPH at a ratio of 1:1 by weight for 2 h at 4 ℃. After the EVs solution is completely absorbed by SPH, the assembly of SPH-EVs is completed. In order to verify the successful assembly of SPH-EVs, the SPH-EVs was observed by scanning electron microscope. According to the results of scanning electron microscope, EVs are uniformly distributed in SPH, which proves that SPH-EVs is successfully assembled (Fig. 5G). The release pattern of SPH-EVs was assessed based on prior research [56, 57]. In summary, 100 µg of SPH-EVs was placed in a transwell chamber within a 24-well plate and incubated with 500 µl of PBS at 37°C. Subsequently, 10 µl of the supernatant was collected and replaced with an equal volume of fresh PBS on specified days. The concentration of released EVs was determined using the BCA Protein Assay Kit (Fig. 5H). The results show that SPH-EVs can release EVs slowly.

#### SPH-EVs has a better effect on promoting tenocytes recovery

**Fig. 6:**
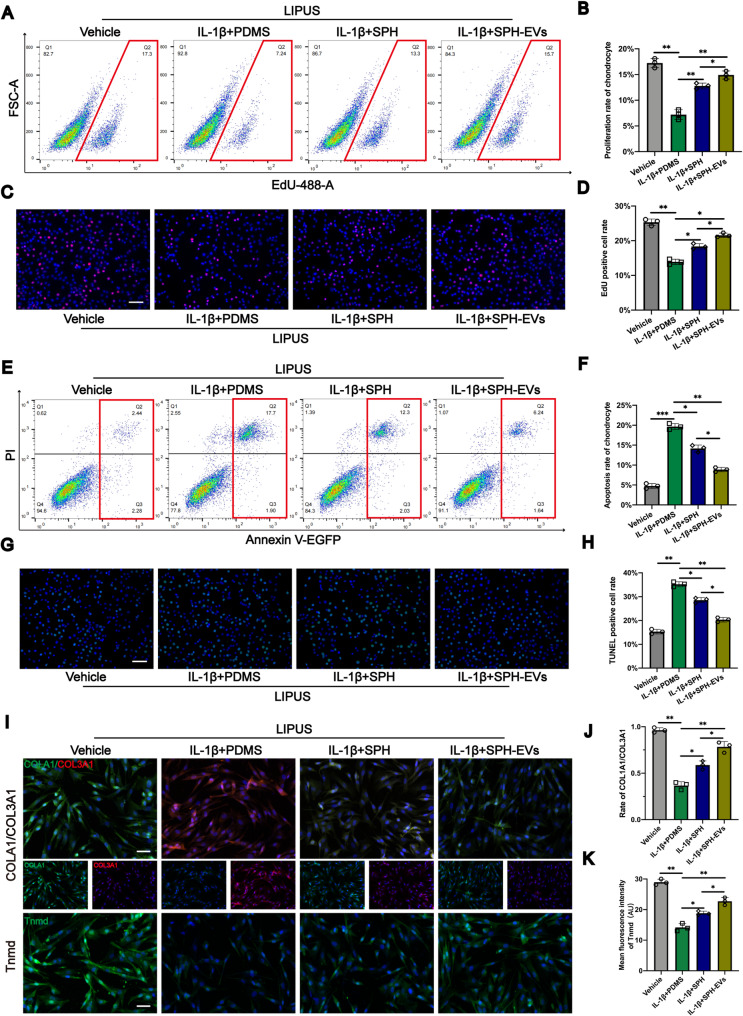
SPH-EVs has a better effect on promoting tenocytes recovery. **A**,** B.** EdU assay for proliferation rate of tenocytes was determined by flow cytometry analysis and the statistical results of flow cytometry analysis for EdU assay (*n* = 3, one-way ANOVA). **C**,** D.** The proliferation rate of tenocytes was observed by immunofluorescence and the EdU positive cell rate was analyzed in each group (*n* = 3, one-way ANOVA) (Scale Bar = 100 μm). **E**,** F.** The Annexin V-FITC/PI Apoptosis assay for apoptosis rate of tenocytes was determined by flow cytometry analysis and the statistical results of flow cytometry analysis for apoptosis analysis (*n* = 3, one-way ANOVA). **G**,** H.** The apoptosis rate of tenocytes was observed by immunofluorescence and the TUNEL positive cell rate was analyzed in each group (*n* = 3, one-way ANOVA) (Scale Bar = 100 μm). **I**,** J and K.** Expression levels of COL1A1, COL3A1 and Tnmd as determined by immunofluorescence staining (Scale Bar = 50 μm). Data are presented as mean ± SD. *P < 0.05, **P < 0.01, ***P < 0.001

Due to the scarcity of relevant literatures on in vitro cell modeling of Achilles tendon injury and the fact that the repair after Achilles tendon injury belongs to aseptic inflammation, we had to refer to the method of modeling aseptic inflammation. The concentration of IL-1β used in the in vitro modeling of many aseptic inflammations is 10ng/ml [58–64]. To verify whether SPH-EVs has better repair effects, we simulated in vivo aseptic inflammation after Achilles tendon rupture by adding IL-1β (10 ng/ml) to cell culture, and stimulated SPH/ SPH-EV to generate electrical signals using low-intensity pulsed ultrasound (LIPUS) to simulate Achilles tendon contraction motion. As described in the methods section, the proliferation rate of tenocytes in each group were measured by cytometry. The cell proliferation rate of tenocytes in SPH-EVs group was significantly higher than that in SPH group (Figs. 6A and B), and this phenomenon was also verified in EdU assay (Figs. 6C and D). Similarly, the apoptosis rate of tenocytes in each group was tested. The flow cytometry results showed that the apoptosis rate of SPH-EVs group was significantly lower than that of SPH group (Figs. 6E and F), and the same result appeared in Tunel assay (Figs. 6G and H). In addition, we also measured the expression of related proteins in each group of tenocytes. The results of cellular immunofluorescence showed that the rate of COL1A1/COL3A1 in SPH-EVs group was significantly higher than that in SPH group (Figs. 6I and J), indicating that SPH-EVs could promote the expression of COL1A1 in tenocytes and inhibit the expression of COL3A1. At the same time, the expression of Tnmd in SPH-EVs group was significantly higher than that in SPH group (Figs. 6I and K). The above results are shown that SPH-EVs has a better effect on promoting tenocytes recovery.

#### SPH-EVs has better function of improving functional behavior and motor ability after Achilles tendon injury in rats

**Fig. 7:**
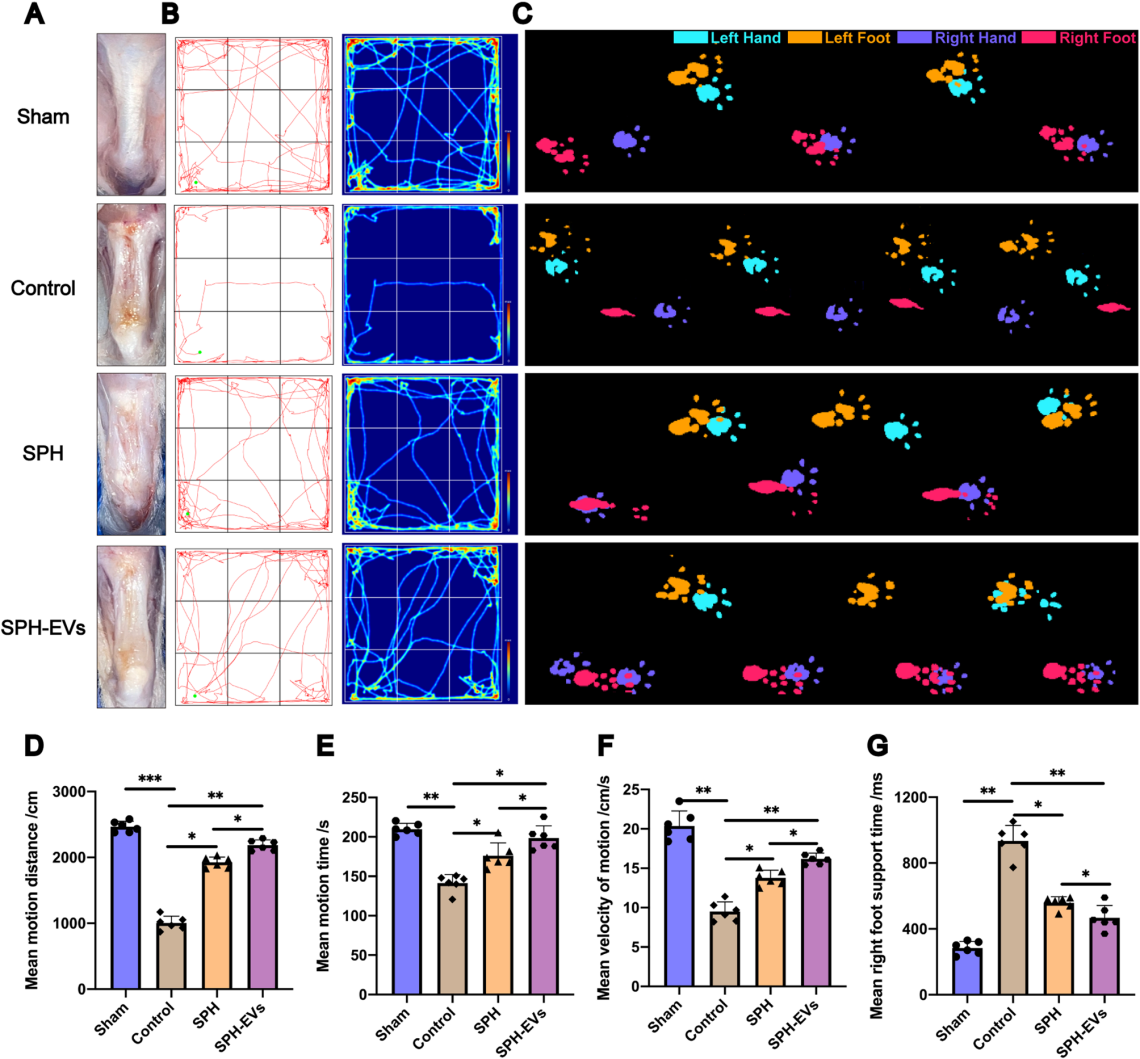
SPH-EVs has better function of improving functional behavior and motor ability after achilles tendon injury in rats. **A**. General view of the tendon in each group after 8 weeks. **B**. Motion trace and heat map of each group rat in open field test. **C**. The results of footprint analysis in each group of rats. **D**, **E**. The mean motion time and distance of rats in open field test (*n* = 6, one-way ANOVA). **F**. The mean motion speede and right foot support time of rats in footprint analysis (*n* = 6, one-way ANOVA). Data are presented as mean ± SD. *P < 0.05, **P < 0.01, ***P < 0.001

In order to better evaluate the repair function of SPH-EVs on Achilles tendon repture, we established a rat Achilles tendon repture model, and used SPH and SPH-EVs to wrap around the injured Achilles tendon respectively. After 8 weeks of modeling, the Achilles tendon was taken for gross morphological evaluation. The results showed that compared with the SPH group, the rats in the SPH-EVs group had less scarring of the Achilles tendon, and the problem of thickening and shortening of the Achilles tendon was significantly improved (Fig. 7A). And the open field test and the footprint analysis were carried out to evaluate the recovery of Achilles tendon in each group from the perspective of behavior. The results of open field test showed that compared with the SPH group, the average moving distance and the average moving time of the SPH-EVs group were better than those of the Control group (Figs. 7B, D and E). Moreover, the results of footprint analysis also showed that the SPH-EVs group showed significantly faster gait recovery and improved motor co-ordination compared with the SPH group (Figs. 7C and F). The mean right foot support time of SPH-EVs group was also significantly lower than that of SPH group (Figs. 7C and E). These results all confirm that, compared with SPH, SPH-EVs has better function of improving functional behavior and motor ability.

#### SPH-EVs has a better function of promoting the repair of Achilles tendon after injury at historical level

**Fig. 8:**
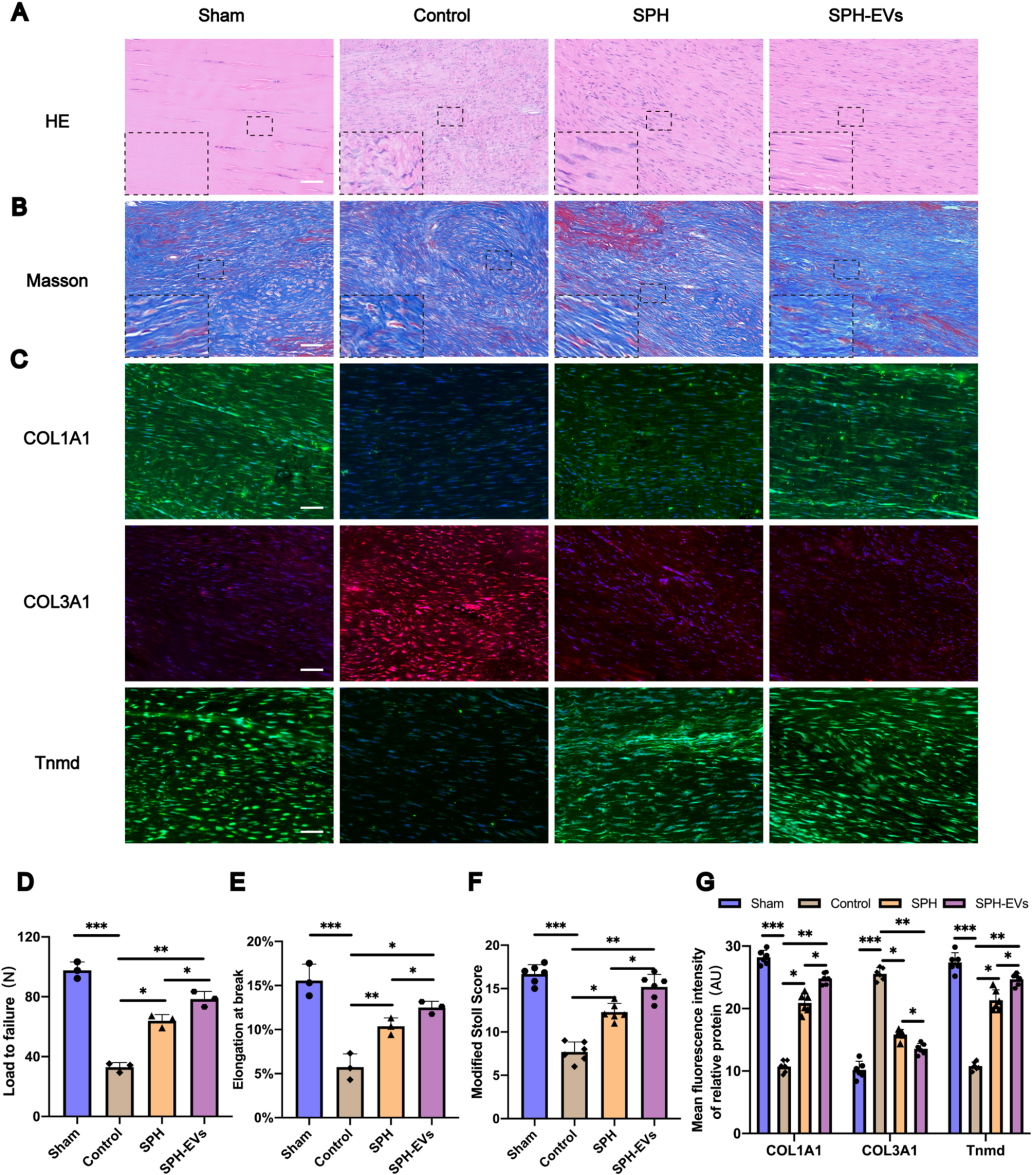
SPH-EVs has a better function of promoting the repair of achilles tendon after injury at historical level. **A**,** B.** HE staining and Masson staining of the tendon in each group after 8 weeks (Scale Bar = 400 μm). **C.** Expression levels of COL1A1, COL3A1 and Tnmd as determined by immunofluorescence staining (Scale Bar = 400 μm). **D.** Load to failure of Achilles tendon tissue in each group (*n* = 3, one-way ANOVA). **E.** Elongation at break of Achilles tendon tissue in each group (*n* = 3, one-way ANOVA). **F.** Quantitative analysis of HE staining according to the modified Stoll score (*n* = 6, one-way ANOVA). **G.** Quantification of mean fluorescence intensity of COL1A1, COL3A1 and Tnmd in each group (*n* = 6, one-way ANOVA). Data are presented as mean ± SD. *P < 0.05, **P < 0.01, ***P < 0.001

After 8 weeks of modeling, we conducted biomechanical analysis of the Achilles tendon samples in each group to evaluate the strength of the Achilles tendon after healing. The results showed that compared with the SPH group, the Achilles tendons in the SPH-EVs group had larger load to failure and elongation at break (Figs. 8D and E), second only to the Sham group, indicating that the Achilles tendons in the SPH-EVs group had greater strength and better recovery. Next, HE staining and Masson staining were performed on the Achilles tendon tissues of each group. Compared with the SPH group, the problems of tendon extracellular matrix collagen arrangement disorder, cell proliferation, scar formation and vascular hyperplasia were significantly improved in the SPH-EVs group (Figs. 8A and B). The modified Stoll score showed higher scores in the SPH-EVs group compared to the SPH group, indicating better healing in the SPH-EVs group (Fig. 8F).

In addition, immunofluorescence staining was also performed on the Achilles tendon of each group to observe the expression of different proteins in the Achilles tendon of each group. Immunofluorescence staining showed that the expression of COL1A1 and Tnmd was higher and the expression of COL3A1 was lower in SPH-EVs group than in SPH group (Figs. 8C and G). All these results indicated that the healing of Achilles tendon in SPH-EVs group was better than that in SPH group.

#### Differences in mRNA sequencing results of rat Achilles tendon tissues from the PDMS group, the SPH group and the SPH-EVs group

In order to explore the potential mechanism of how SPH promotes tendon regeneration, we conducted mRNA sequencing on the Achilles tendons of rats in the SPH group and the PDMS group. Using a criteria of fold change > 2 and q-value < 0.05, a total of 1284 differentially expressed mRNAs were identified, including 423 upregulated mRNAs and 861 downregulated mRNAs. The specific distribution is shown in a volcano plot (Supplementary Fig. 3A). We selected genes with more significant differences and displayed them in a heatmap (Supplementary Fig. 3B). To further understand the biological processes that differentially expressed mRNAs may mediate, we performed gene ontology enrichment analysis on the sequencing results. The analysis was conducted from perspectives Biological process, Cellular component and Molecular function, and the results were presented in the form of histograms (Supplementary Figs. 3C, D and E). Additionally, we also conducted analysis from perspective Statistics of Pathway Enrichment, with specific results shown in a bubble chart (Supplementary Fig. 3F). Furthermore, based on the mRNA sequencing results, we detected several genes highly related to tendon regeneration. The results showed that compared to the PDMS group, the expression of genes related to COL1A1 and COL3A1 significantly increased in the SPH group, while the expression of genes related to COL2A1 decreased. Genes related to matrix metalloproteinases (MMPs) and tendon-related gene Mmp3 showed decreased expression, while Mmp9, Mmp13, Tnmd, and Scx had significantly increased expression in the SPH group (Supplementary Fig. 3G).

To explore the potential mechanism by which SPH-EVs promote tendon regeneration, we conducted mRNA sequencing on the Achilles tendons of rats in the SPH-EVs group and the PDMS group. Using a standard of fold change > 2 and q-value < 0.05, a total of 2236 mRNAs with differential expression were identified, including 732 upregulated mRNAs and 1504 downregulated mRNAs. The specific distribution is shown in a volcano plot (Supplementary Fig. 4A). We extracted genes with more significant differences and displayed them in a heatmap (Supplementary Fig. 4B). To gain a deeper understanding of the biological processes that differentially expressed mRNAs may mediate, we conducted gene ontology enrichment analysis on the sequencing results from perspectives Biological process, Cellular component, and Molecular function, with the results presented in the form of histograms (Supplementary Figs. 4C, D and E). Moreover, we also conducted analysis from perspective Statistics of Pathway Enrichment, with specific results shown in a bubble chart (Supplementary Fig. 4F). In addition, based on the mRNA sequencing results, we detected several genes highly related to tendon regeneration. The results showed that compared to the PDMS group, the expression of genes related to COL1A1 and COL3A1 significantly increased in the SPH-EVs group, while the expression of genes related to COL2A1 decreased. Furthermore, the expression of Mmp-related genes and tendon-related gene Mmp3 decreased, while Mmp9, Mmp13, Tnmd, and Scx showed significantly increased expression in the SPH-EVs group (Supplementary Fig. 4G).

**Fig. 9:**
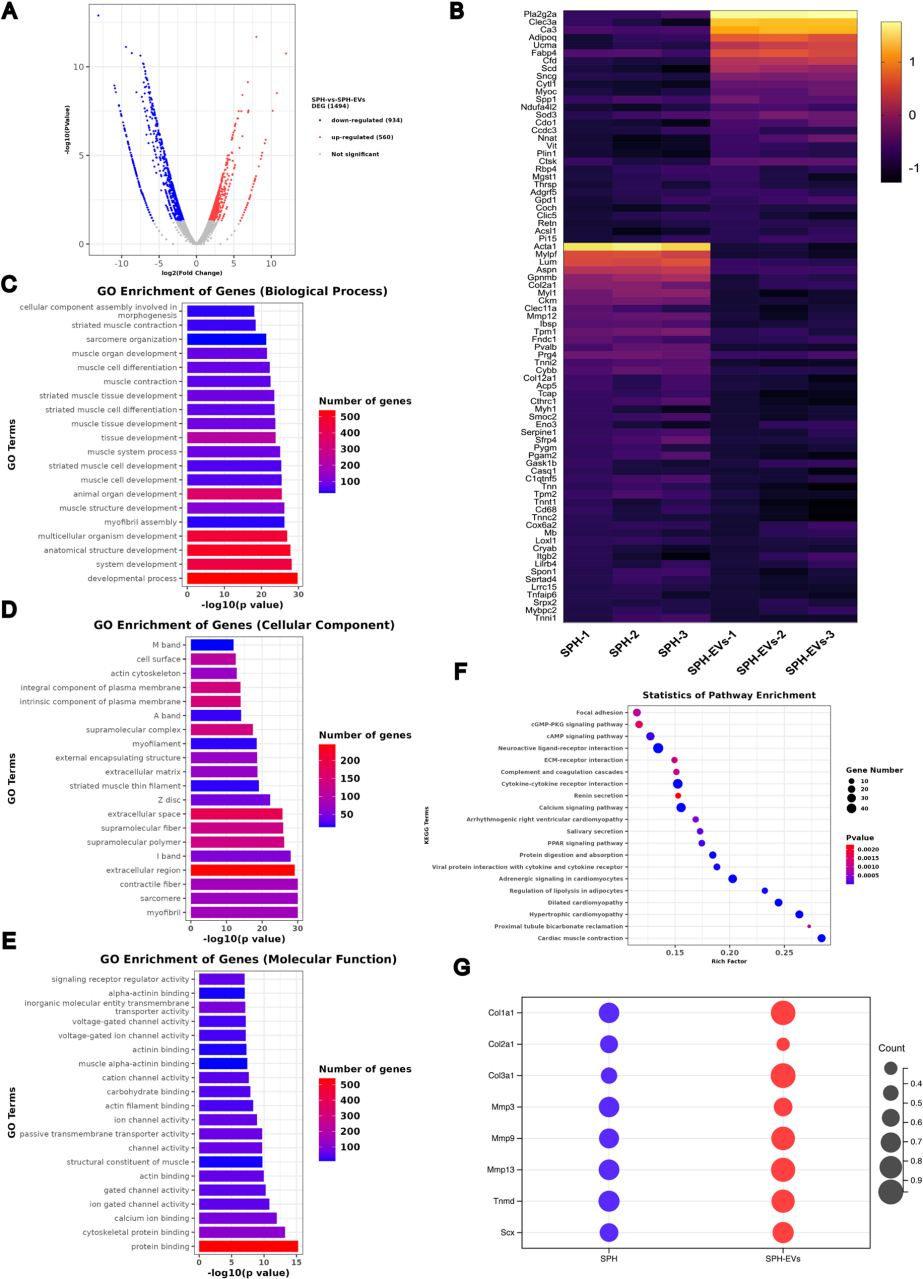
RNA-sequencing analysis of differentially expressed genes between the SPH and SPH-EVs group. **A**. Volcano plot of differentially expressed genes between the SPH and SPH-EVs group. **B**. Heat maps show differences in gene expression between SPH and SPH-EVs group. **C**. GO enrichment analysis showed differences between SPH and SPH-EVs group (Biological Process). **D**. GO enrichment analysis showed differences between SPH and SPH-EVs group (Celluar Component). **E**. GO enrichment analysis showed differences between SPH and SPH-EVs group (Molecular Function). **F**. The results of statistics of pathway enrichment show the difference between SPH and SPH-EVs group. **G**. Relative expression of specific genes between the SPH and SPH-EVs group

To explore the potential mechanism by which SPH-EVs promote tendon repair, we conducted mRNA sequencing on the Achilles tendons of rats in the SPH-EVs group and the SPH group. Using a criteria of fold change > 2 and q-value < 0.05, a total of 1494 mRNAs with differential expression were identified, including 560 upregulated mRNAs and 934 downregulated mRNAs. The specific distribution is shown in a volcano plot (Fig. 9A). We selected genes with more significant differences and displayed them in a heatmap (Fig. 9B). To gain a deeper understanding of the biological processes that differentially expressed mRNAs may mediate, we performed gene ontology enrichment analysis on the sequencing results. The analysis was conducted from perspectives Biological process, Cellular component and Molecular function, with the results presented in the form of histograms (Figs. 9C, D and E). Additionally, we also conducted analysis from perspective Statistics of Pathway Enrichment, with specific results shown in a bubble chart (Fig. 9F). Furthermore, based on the mRNA sequencing results, we detected several genes highly related to tendon regeneration. The results showed that compared to the PDMS group, the expression of genes related to COL1A1 and COL3A1 significantly increased in the SPH-EVs group, while the expression of genes related to COL2A1 decreased. Moreover, the expression of Mmp-related genes and tendon-related gene Mmp3 decreased, while Mmp9, Mmp13, Tnmd, and Scx showed significantly increased expression in the SPH-EVs group (Fig. 9G).

The above results show the effect of SPH and SPH-EVs on rat Achilles tendon mRNA, and the results corroborate with the results of the biological part above, which provide direction and guidance for us to further investigate the biological mechanism of SPH and SPH-EVs on Achilles tendon repair and regeneration.

### Discussion

Achilles tendon rupture injuries, one of the most common musculoskeletal injuries, remain a key challenge in the field of orthopedic surgery [65]. More innovative solutions are needed to overcome the limitations of current surgical treatments for Achilles tendon rupture injuries. Bioelectronic therapies have shown great potential in the treatment of musculoskeletal disorders by accelerating functional recovery through the activation of tissue regeneration-specific signaling pathways [66–69]. Among the many self-powered bioelectronic devices, piezoelectric materials offer a completely new direction for the treatment of Achilles tendon rupture injuries due to their lack of need for batteries or external power supply and their extreme biocompatibility and plasticity. In this study, by mixing BQPR elastomer with sodium alginate in a certain proportion, SPH with piezoelectricity and water absorption of hydrogel material was obtained through a series of operations. Compared with other piezoelectric materials, BQPR elastomers are non-toxic, biocompatible and low-cost, and are derived from plants such as corn cobs and stalks where biobased monomers can be extracted. Not only that, in the entire cycle of BQPR, only water is involved, non-toxic and harmless, and it is safer as a material implanted in the human body. In addition, the elastic modulus of SPH is similar to that of the Achilles tendon tissue, and it has good ductility and piezoelectric property. It can be said that the mechanical energy of the Achilles tendon tissue can be well converted into electrical energy, thus stimulating the regeneration of the Achilles tendon tissue.

Previous studies have demonstrated the great potential of MSCs transplantation for the treatment of various degenerative diseases and injuries [10–12]. However, the direct application of MSCs has been limited because direct transplantation of MSCs may lead to chromosomal mutations and immune rejection as side effects [13, 14]. Surprisingly, the direct use of extracellular vesicles derived from MSCs can perfectly circumvent the above mentioned side effects and at the same time can have a corresponding therapeutic effect on the injury site [18, 19]. Several studies have demonstrated that the use of MSCs-derived extracellular vesicles can significantly promote the repair of Achilles tendon injuries [44, 70–72]. At the same time, many studies have demonstrated that hydrogels loaded with extracellular vesicles have achieved surprising therapeutic results in the treatment of wound healing [73, 74], bone defect repair [75, 76], cartilage repair [77, 78], and repair after nerve injury [79, 80]. SPH, as a novel piezoelectric hydrogel, has both the piezoelectricity of piezoelectric materials and at the same time the high water absorption and degradability of hydrogels. In the field of Achilles tendon rupture injury repair, there has never been a precedent of combining piezoelectric materials with extracellular vesicles derived from mesenchymal stem cells, so we made a bold attempt in this direction and obtained SPH-EVs. Due to the high water absorbency of SPH and its completely degradable properties, we obtained SPH-EVs by incorporating SPH into the PBS solution containing EVs. However, due to the rapid clearance of body fluids and circulation, it is difficult to release extracellular vesicles continuously and slowly at a specific site, and therefore it is necessary to continuously replenish extracellular vesicles at the affected site by injection to achieve optimal therapeutic effect [24, 25]. While slowly degrading SPH in the body, SPH-EVs continuously releases EVs at the Achilles tendon rupture site, avoiding the pain caused to the patient by multiple invasive replenishment of EVs at the affected site. In the rat Achilles tendon rupture model, compared with the SPH group, the SPH-EVs group had less scar tissue formation, better mechanical and behavioral performance. All these indicate that SPH-EVs has better Achilles tendon repair ability.

After Achilles tendon surgery, due to the invasive nature of the surgery and the long recovery time, secondary rupture of the Achilles tendon occurs in about 5% of patients who undergo rehab without monitoring of Achilles tendon movement intensity [4–7]. Patients with secondary Achilles tendon ruptures are more difficult to operate on, have a longer recovery time, and have more functional deficits in the Achilles tendon after recovery. In order to solve this problem, we combine the NFC module that can monitor the voltage in real time with SPH-EVs, so as to achieve real-time monitoring of the movement intensity of the Achilles tendon tissue. The specific principle is that different intensity of movement will make SPH produce different intensity of voltage, NFC module can convert mechanical signals into electrical signals, real-time transmission to the display, so that the patient’s rehabilitation more safe, reduce the risk of Achilles tendon re-rupture.

In summary, in this study, we innovatively integrated sodium alginate with BQPR piezoelectric elastomer to develop a novel piezoelectric hydrogel material that exhibits excellent water absorption and biocompatibility. This advancement significantly addresses the issue of low biocompatibility observed in previous piezoelectric materials. Owing to its superior water absorption capacity, SPH can effectively encapsulate a large quantity of EVs. Upon implantation in vivo, its slow degradation property enables the continuous and controlled release of EVs at the Achilles tendon fracture site, ensuring therapeutic efficacy without the need for repeated invasive supplementation. This not only alleviates patient discomfort but also minimizes the risk of infection. Furthermore, experimental results demonstrated that the combination of electrical stimulation and EVs yielded substantially better outcomes in repairing ruptured Achilles tendons compared to either approach alone. Additionally, the NFC module embedded in SPH-EVs allows for real-time monitoring of the voltage at the Achilles tendon fracture site, thereby guiding patients to perform rehabilitation exercises within a safe range. This dual-functionality helps restore tendon function while mitigating the risk of re-fracture caused by excessive rehabilitation. In conclusion, this study highlights the potential of SPH-EVs in both monitoring tendon status and promoting tendon regeneration, paving the way for future clinical applications.

## Conclusions

This study reveals that the combination of EVs with smart piezoelectric hydrogels SPH significantly enhances the healing process of Achilles tendon ruptures. The electrical stimulation produced by the piezoelectric effect, along with the gradual release of vesicles, not only speeds up tissue repair but also minimizes scar formation and improves the tendon’s mechanical strength. Furthermore, the integrated NFC module allows for real-time monitoring of exercise intensity, helping patients maintain appropriate activity levels during rehabilitation and lowering the risk of re-rupture. All the above results prove that SPH-EVs meets the hypothesis we made at the beginning of designing materials. This innovative approach offers fresh insights into the treatment of Achilles tendon injuries, greatly improving the recovery experience and outcomes for patients.

## Electronic supplementary material

Below is the link to the electronic supplementary material.

Supplementary Material 1

## Data Availability

No datasets were generated or analysed during the current study.
