# Supplementary material for: Sodium alginate piezoelectric hydrogel loaded with extracellular vesicles derived from bone marrow mesenchymal stem cells promotes repair of Achilles tendon rupture
Source: J Nanobiotechnology. 2025 Oct 1;23:625. doi: 10.1186/s12951-025-03606-5 (PMC12486638; doi:10.1186/s12951-025-03606-5)
Supplement: Supplementary file 1 — Supplementary Material 1 [file 12951_2025_3606_MOESM1_ESM.docx]

**Supplementary Figure**


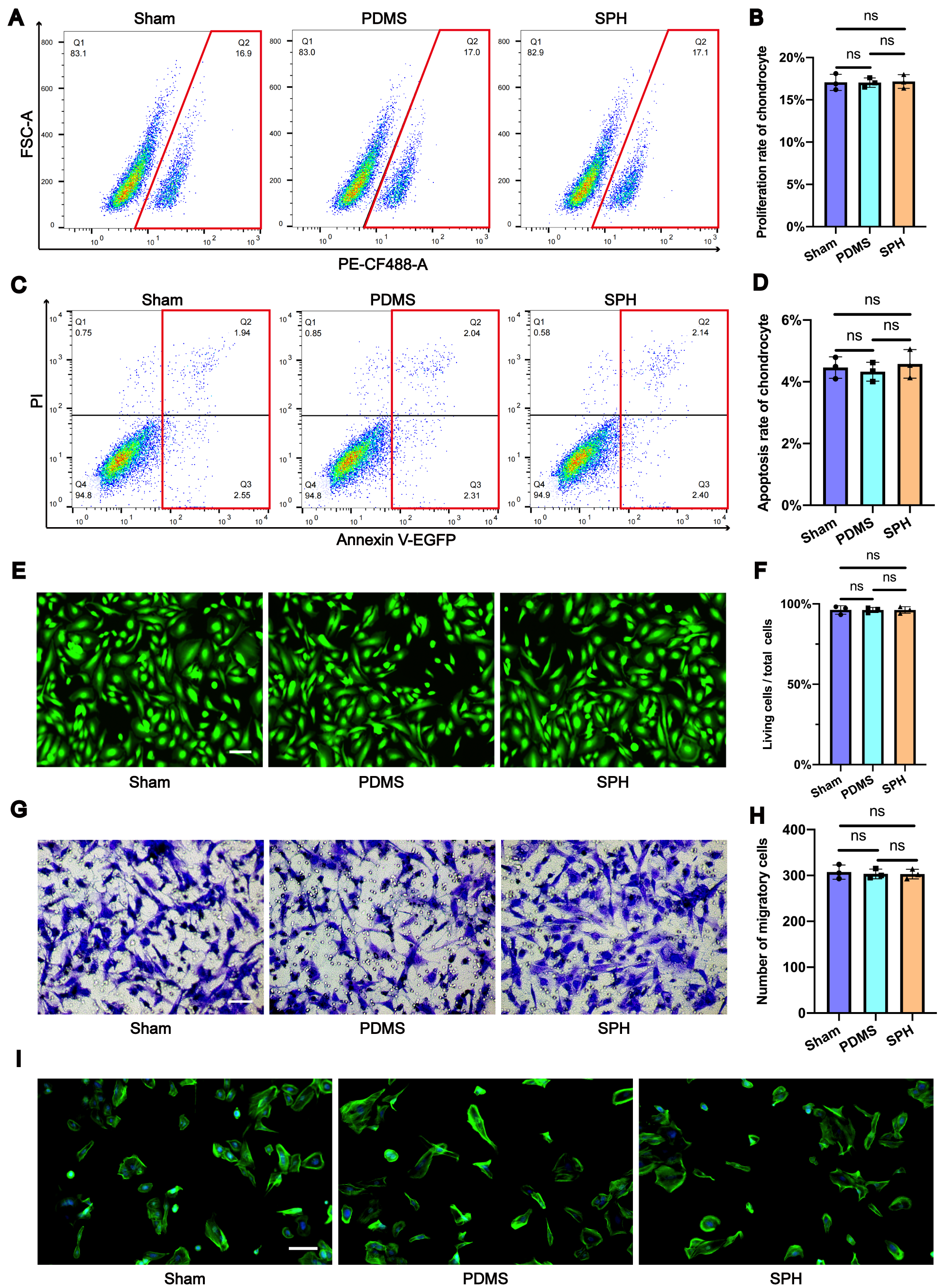


**Supplementary Fig.1 In vitro experiments proved that SPH had excellent biocompatibility with rat tenocytes. A,B.** EdU assay for proliferation rate of tenocytes was determined by flow cytometry analysis and the statistical results of flow cytometry analysis for EdU assay (n = 3, one-way ANOVA). **C,D.** The Annexin V-FITC/PI Apoptosis assay for apoptosis rate of tenocytes was determined by flow cytometry analysis and the statistical results of flow cytometry analysis for apoptosis analysis(n = 3, one-way ANOVA). **E,F.** Dead and live assay evaluation of tenocytes proliferation and the statistical results of dead and live assay(n = 3, one-way ANOVA).(Scale Bar = 100 μm). **G,H.** Migration of tenocytes was observed and quantified by transwell assay and crystal violet was used to stain migrating tenocytes and the statistical results of transwell assay(n = 3, one-way ANOVA).(Scale Bar = 100 μm). **I.** Results of staining of tenocytes with phalloidine(Scale Bar = 50 μm). Data are presented as mean ± SD. Ns means non-significant.


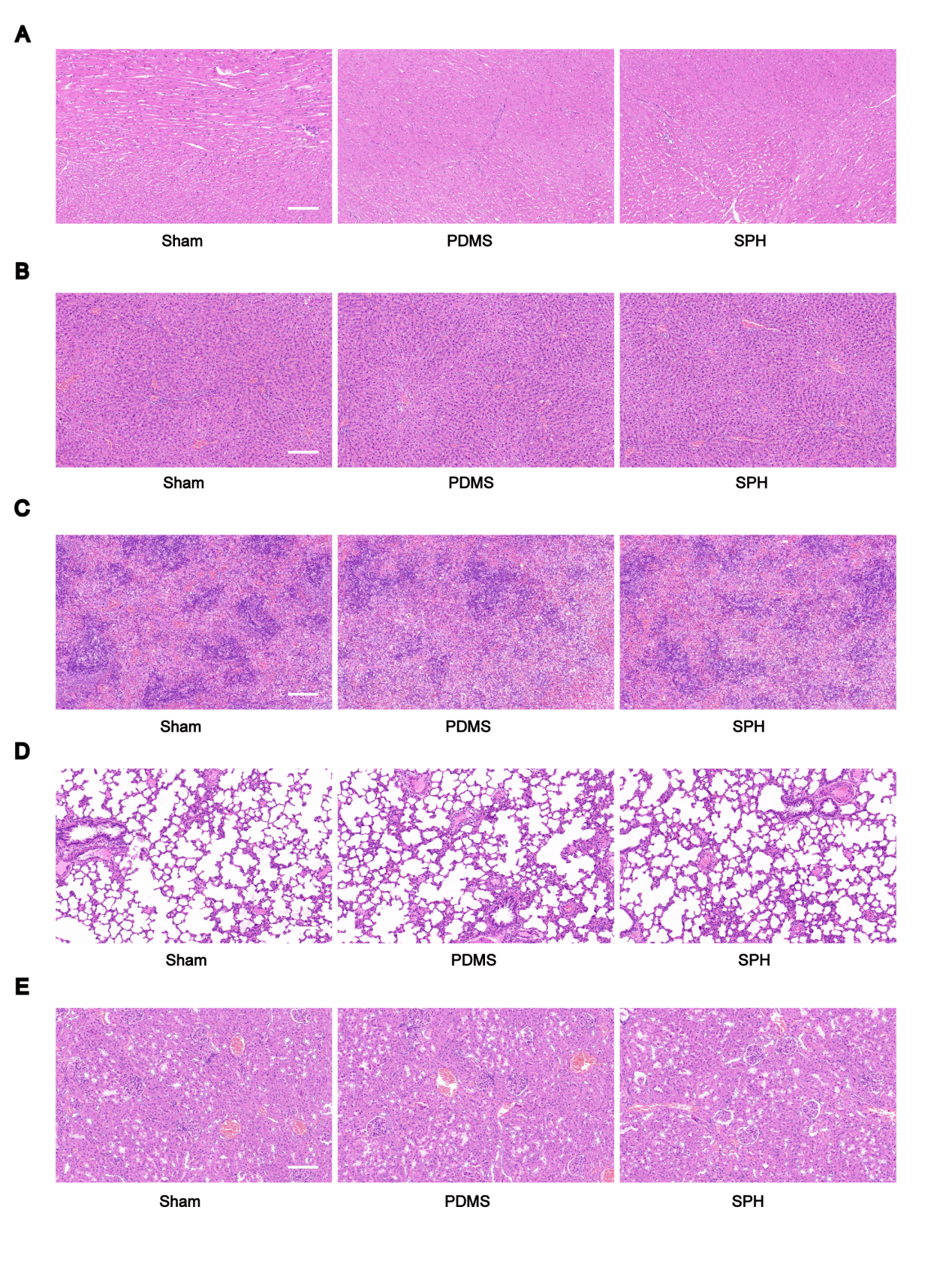


**Supplementary Fig.2 In vivo experiments proved that SPH had excellent biocompatibility. A.** HE staining of heart tissue in each group(Scale Bar = 400 μm). **B.** HE staining of liver tissue in each group(Scale Bar = 400 μm). **C.** HE staining of spleen tissue in each group(Scale Bar = 400 μm). **D.**HE staining of lung tissue in each group(Scale Bar = 400 μm). **E.** HE staining of kidney tissue in each group(Scale Bar = 400 μm).
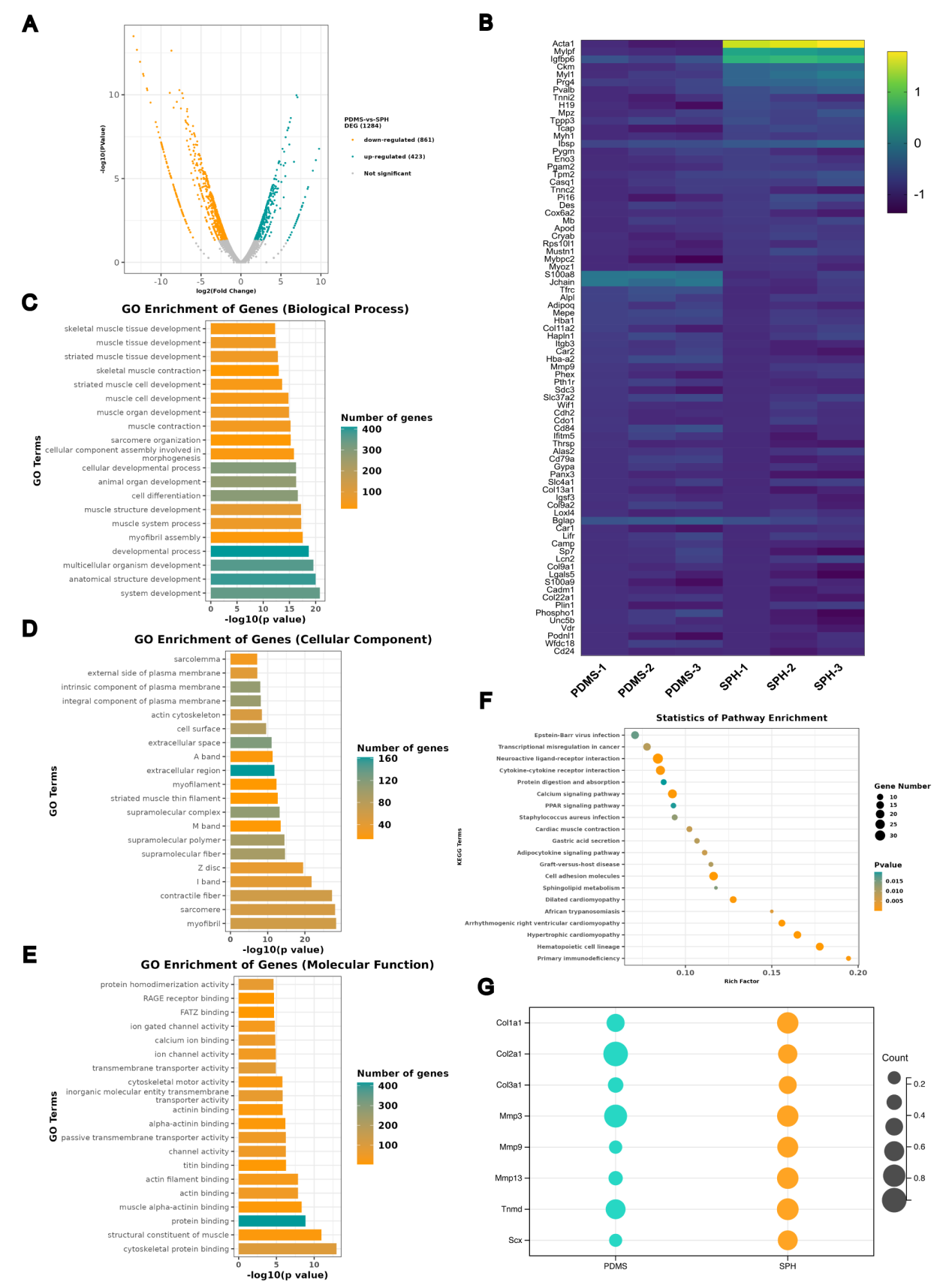


**Supplementary Fig.3 RNA-sequencing analysis of differentially expressed genes between the PDMS and SPH group. A.** Volcano plot of differentially expressed genes between the PDMS and SPH group. **B.** Heat maps show differences in gene expression between PDMS and SPH group. **C.** GO enrichment analysis showed differences between PDMS and SPH group(Biological Process). **D.** GO enrichment analysis showed differences between PDMS and SPH group(Celluar Component). **E.** GO enrichment analysis showed differences between PDMS and SPH group(Molecular Function). **F.** The results of statistics of pathway enrichment show the difference between PDMS and SPH group. **G.** Relative expression of specific genes between the PDMS and SPHgroup.
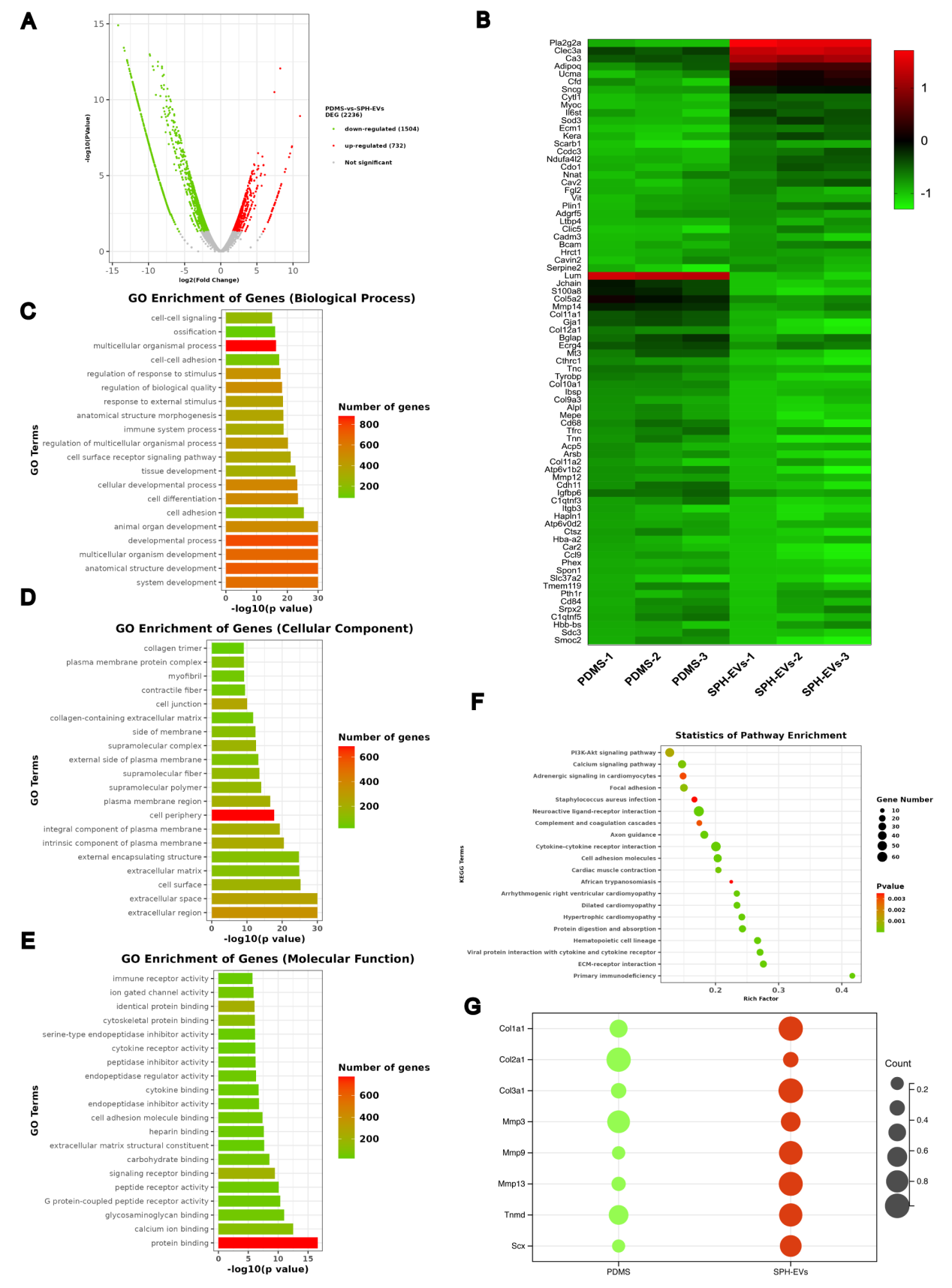


**Supplementary Fig.4 RNA-sequencing analysis of differentially expressed genes between the PDMS and SPH-EVs group. A.** Volcano plot of differentially expressed genes between the PDMS and SPH-EVs group. **B.** Heat maps show differences in gene expression between PDMS and SPH-EVs group. **C.** GO enrichment analysis showed differences between PDMS and SPH-EVs group(Biological Process). **D.** GO enrichment analysis showed differences between PDMS and SPH-EVs group(Celluar Component). **E.** GO enrichment analysis showed differences between PDMS and SPH-EVs group(Molecular Function). **F.** The results of statistics of pathway enrichment show the difference between PDMS and SPH-EVs group. **G.** Relative expression of specific genes between the PDMS and SPH-EVs group.
